# Comparative transcriptome analysis and ChIP-sequencing reveals stage-specific gene expression and regulation profiles associated with pollen wall formation in *Brassica rapa*

**DOI:** 10.1186/s12864-019-5637-x

**Published:** 2019-04-03

**Authors:** Xiuping Shen, Liai Xu, Yanhong Liu, Heng Dong, Dong Zhou, Yuzhi Zhang, Sue Lin, Jiashu Cao, Li Huang

**Affiliations:** 1 Laboratory of Cell & Molecular Biology, Institute of Vegetable Science, Zhejiang University, Hangzhou, 310058 China; 2 Key Laboratory of Horticultural Plant Growth, Development and Quality Improvement, Ministry of Agriculture / Zhejiang Provincial Key Laboratory of Horticultural Plant Integrative Biology, Hangzhou, 310058 China; 3 Institute of Life Sciences, Wenzhou University, Wenzhou, 325000 China

**Keywords:** *Brassica rapa*, Male sterility, Pollen, Cytokinesis, Tapetum, Pollen wall, Transcription factor, Transcriptome, ChIP-sequencing

## Abstract

**Background:**

Genic male sterility (GMS) line is an important approach to utilize heterosis in *Brassica rapa*, one of the most widely cultivated vegetable crops in Northeast Asia. However, the molecular genetic mechanisms of GMS remain to be largely unknown.

**Results:**

Detailed phenotypic observation of ‘Bcajh97-01A/B’, a *B. rapa* genic male sterile AB line in this study revealed that the aberrant meiotic cytokinesis and premature tapetal programmed cell death occurring in the sterile line ultimately resulted in microspore degeneration and pollen wall defect. Further gene expression profile of the sterile and fertile floral buds of ‘Bcajh97-01A/B’ at five typical developmental stages during pollen development supported the result of phenotypic observation and identified stage-specific genes associated with the main events associated with pollen wall development, including tapetum development or functioning, callose metabolism, pollen exine formation and cell wall modification. Additionally, by using ChIP-sequencing, the genomic and gene-level distribution of trimethylated histone H3 lysine 4 (H3K4) and H3K27 were mapped on the fertile floral buds, and a great deal of pollen development-associated genes that were covalently modified by H3K4me^3^ and H3K27me^3^ were identified.

**Conclusions:**

Our study provids a deeper understanding into the gene expression and regulation network during pollen development and pollen wall formation in *B. rapa*, and enabled the identification of a set of candidate genes for further functional annotation.

**Electronic supplementary material:**

The online version of this article (10.1186/s12864-019-5637-x) contains supplementary material, which is available to authorized users.

## Background

Anther and pollen development in plants is a complex process which includes a series of extraordinary events such as anther cell division and differentiation, male meiosis, microspores being released from the tetrads, pollen wall development as well as pollen maturation and anther dehiscence. Any abnormality during this process will lead to male sterility, a trait utilized for hybrid breeding and crop yield increases [1].

As a special structure of mature pollen grain, the pollen wall is important for reproduction not only because it provides protection for male gametophytes, but more importantly, because it functions in male-female interaction, fertilization, and seed production [2]. The complex multi-layered pollen wall displays a variety of surface morphologies but a generally similar fundamental structure, comprised of an outer exine and an inner intine [3]. The exine, whose synthesis is regulated by both the sporophytic tapetum and the microspore [4], is constructed primarily of sporopollenin, a robust biopolymer comprised predominantly of polyhydroxylated aliphatic compounds and phenolics [5]. The intine, which is initiated during the early stages of male gametogenesis and is controlled gametophytically [6], is consisting of pectin, cellulose, hemicellulose, hydrolytic enzymes, hydrophobic proteins. As the major event after microspore release from the tetrad, the entire dynamic complex and well-coordinated process of pollen wall development requires the precise spatial and temporal cooperation of gametophytic and sporophytic tissues and metabolic events [7–9]. Recently, well-characterized genes in which mutations cause impaired exine and male sterile phenotype have enriched our understanding in pollen wall development [4, 8, 10, 11]. One of the most striking discoveries is the conversed exine regulation pathway that formed by five transcription factors, DYT1, TDF1, AMS, MYB80, and MS1. This pathway regulates tapetum development and function and thereby influences the developing microspores by controlling callose dissolution, pollen exine formation and tapetal programmed cell death [12–17]. Many other genes, such as lipid transfer protein family members and genes related to lipid and phenolic metabolism are involved in pollen exine formation [18–28]. Previous studies on male sterility focusing on pectin degrading enzymes such as pectin methylesterases (PMEs), polygalacturonases (PGs) and pectate lyases like proteins (PLLs) have also emphasized the important roles of cell wall modification-related genes during the regulation of intine formation and male fertility [29–32]. However, the molecular mechanisms underlying pollen wall patterning remain largely elusive.

Genic male sterility (GMS) is a main type of crop male sterility. In our previous study, a Chinese cabbage (*B. rapa* ssp. *chinensis* cv. Aijiaohuang) genic male sterile A/B line system, named as ‘Bcajh97-01A/B’, was established. The progenies of the A/B line were segregated into sterile and fertile types during reproduction at a 1:1 ratio. In the sterile plant, an aberrant meiotic cytokinesis at the early pollen developmental stage resulted in the degeneration of microspore content and consequently the formation of aborted mature pollen grains only coated with a defective exine wall [33, 34]. Thus, this GMS system serves as a good material to study the pollen wall formation and pollen development.

To better understand the mechanism of pollen wall development and male sterility, in this work, using the sterile and fertile floral buds of ‘Bcajh97-01A/B’ as materials, a detailed gene expression profile at five typical developmental stages, namely, pollen mother cells, tetrad, uninucleate pollen, binucleate pollen, and mature pollen stage, were further examined by RNA sequencing (RNA-seq). Together with the detailed phenotype investigation on the difference of dynamic pollen callose change and tapetum degradation between the sterile and fertile anther, candidate genes that associate with these two developmental events were identified. What’s more, by using ChIP-sequencing (ChIP-seq), we mapped the genomic and gene-level distribution of trimethylated histone H3 lysine 4 (H3K4) and H3K27, two histone modifications associated with gene activation and silencing, respectively, on the fertile floral buds, to explore the epigenetic control on gene expression during pollen development. These studies provided a deeper understanding into the gene expression network during pollen wall development process in *B. rapa*, and enabled the identification of a set of candidate genes for further functional annotation.

## Results

### Phenotypic characterization of anther and pollen development in the fertile and sterile lines

**Fig. 1:**
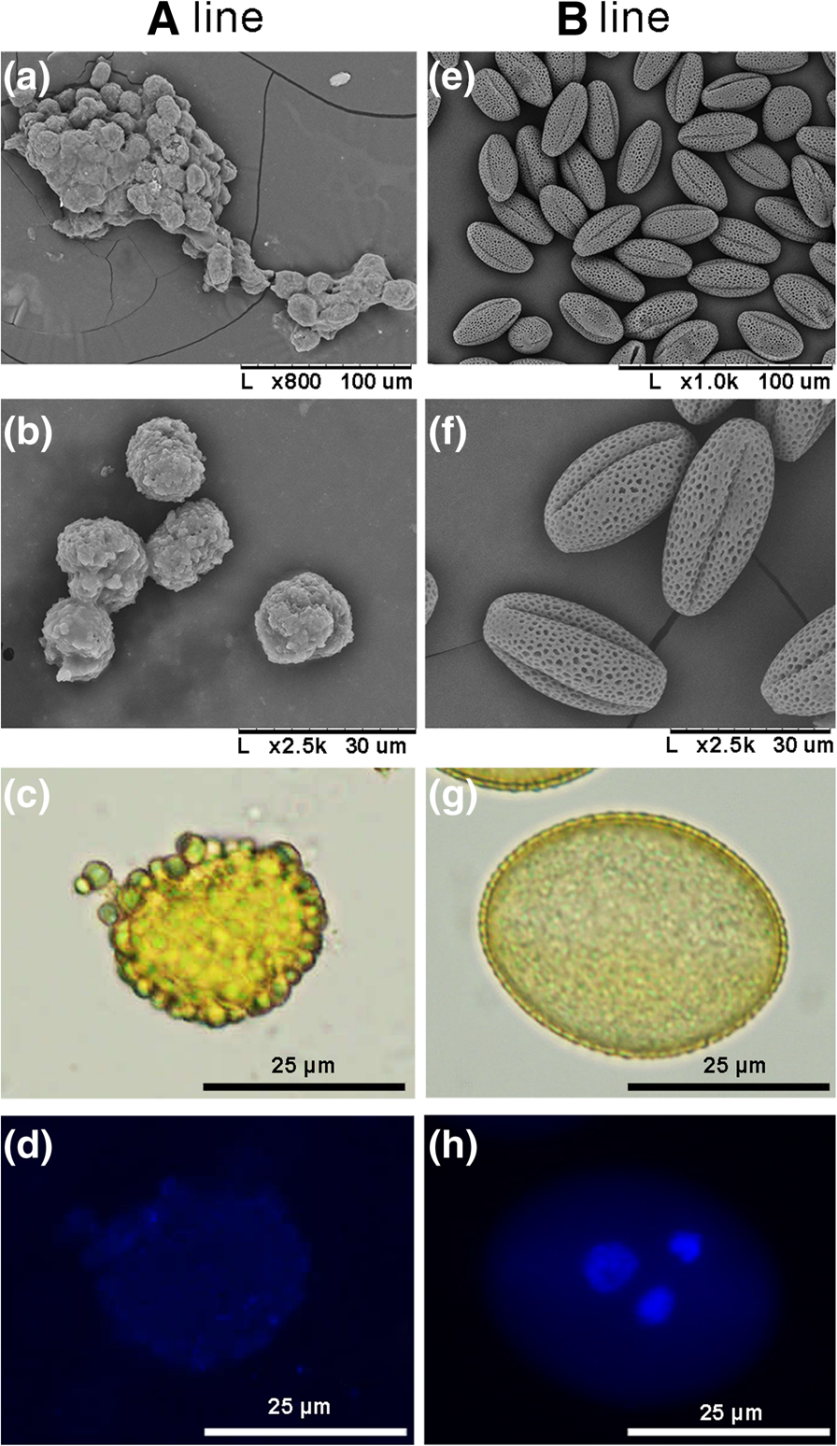
Comparison of pollen grains of the sterile line ‘Bcajh97-01A’ (A line) and the fertile line ‘Bcajh97-01B’ (B line) of *Brassica rapa*. **a**, **e** Scanning electron micrographs of mature pollen grains. **b**, **f** Magnified images show details of the pollen morphology. **c**, **g** Pollen morphology under bright field microscopy. **d**, **h** Pollen grains stained with 4′, 6-diamidino-2-phenylindole (DAPI) solution

**Fig. 2:**
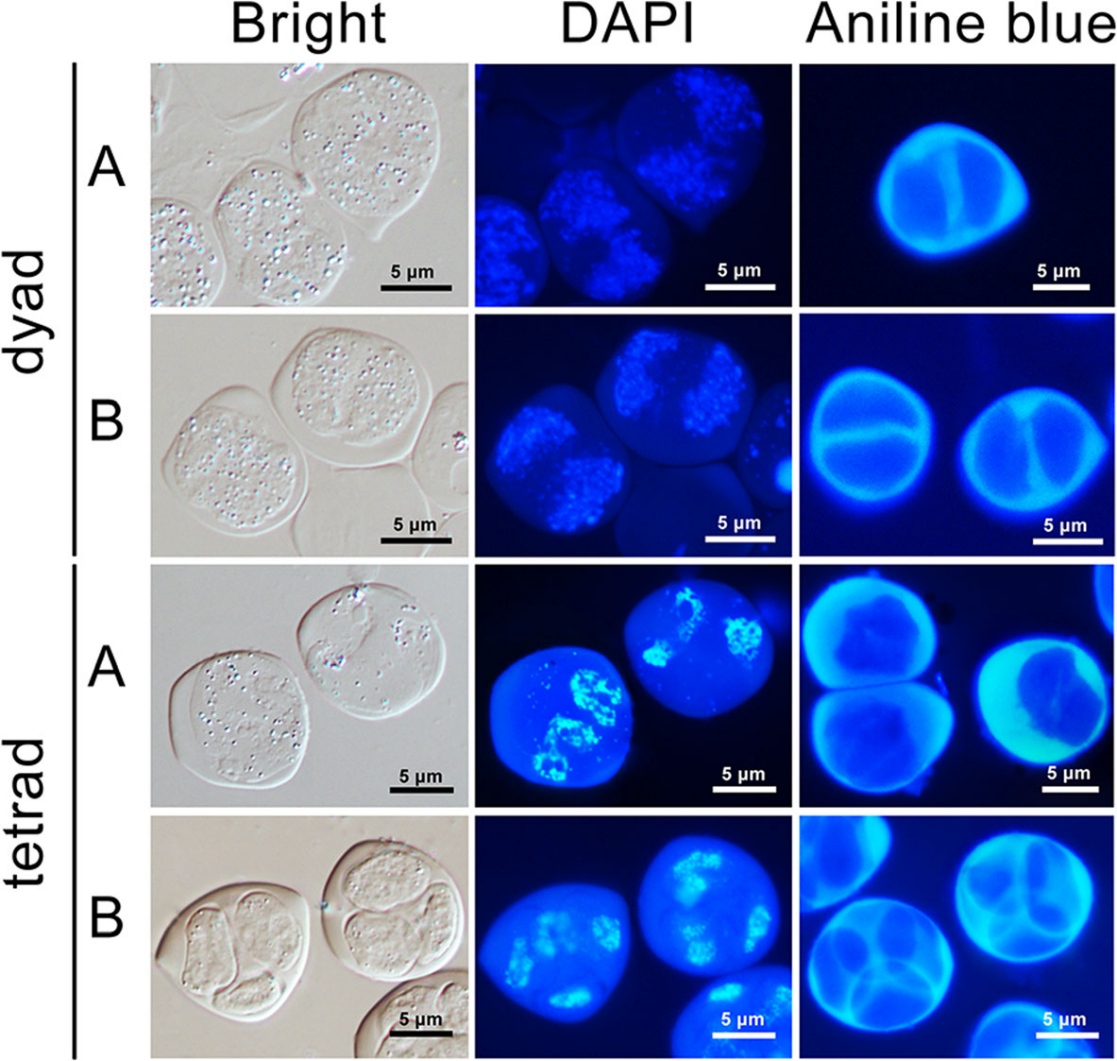
Cytochemical staining for chromatin with DAPI and for callose with aniline blue during the meiosis process of the sterile line ‘Bcajh97-01A’ (A line) and the fertile line ‘Bcajh97-01B’ (B line) of *Brassica rapa*

The previous morphological observation showed the only difference between the sterile and fertile lines was that the anthers of the sterile line lacked normal mature pollen grains [33]. Here, scanning electron microscopy (SEM) showed that globular remnants with rough surface layers filled in pollen sac of the sterile anther instead of ellipsoidal pollen grains with reticulate exine structure and distinct apertures filling in that of the fertile anther (Fig. 1b and f). Brightfield microscopy revealed that numerous yellowish oily droplets were deposited on the surface of the globular remnants, demonstrating abnormal accumulation of exine-held materials in the sterile anther (Fig. 1c and g). The 4′, 6-diamidino-2-phenylindole (DAPI) staining showed no nucleus formed in the globular remnants (Fig. 1d and h). Previous cytological observation have determined that the aberrant cytokinesis at the end of meiosis caused failure of tetrads formation leading to the male sterility of the sterile line [34]. Here, aniline blue staining showed that the dyads of the sterile and fertile lines have identical callose deposition at the periphery and in the cell plate. However, in contrast to the normally deposited callose in the cell plate of tetrads in the fertile line, the intersporal walls were absent in the tetrads of the sterile line, while, the karyokinesis was demonstrated to be normal by DAPI staining (Fig. 2).

**Fig. 3:**
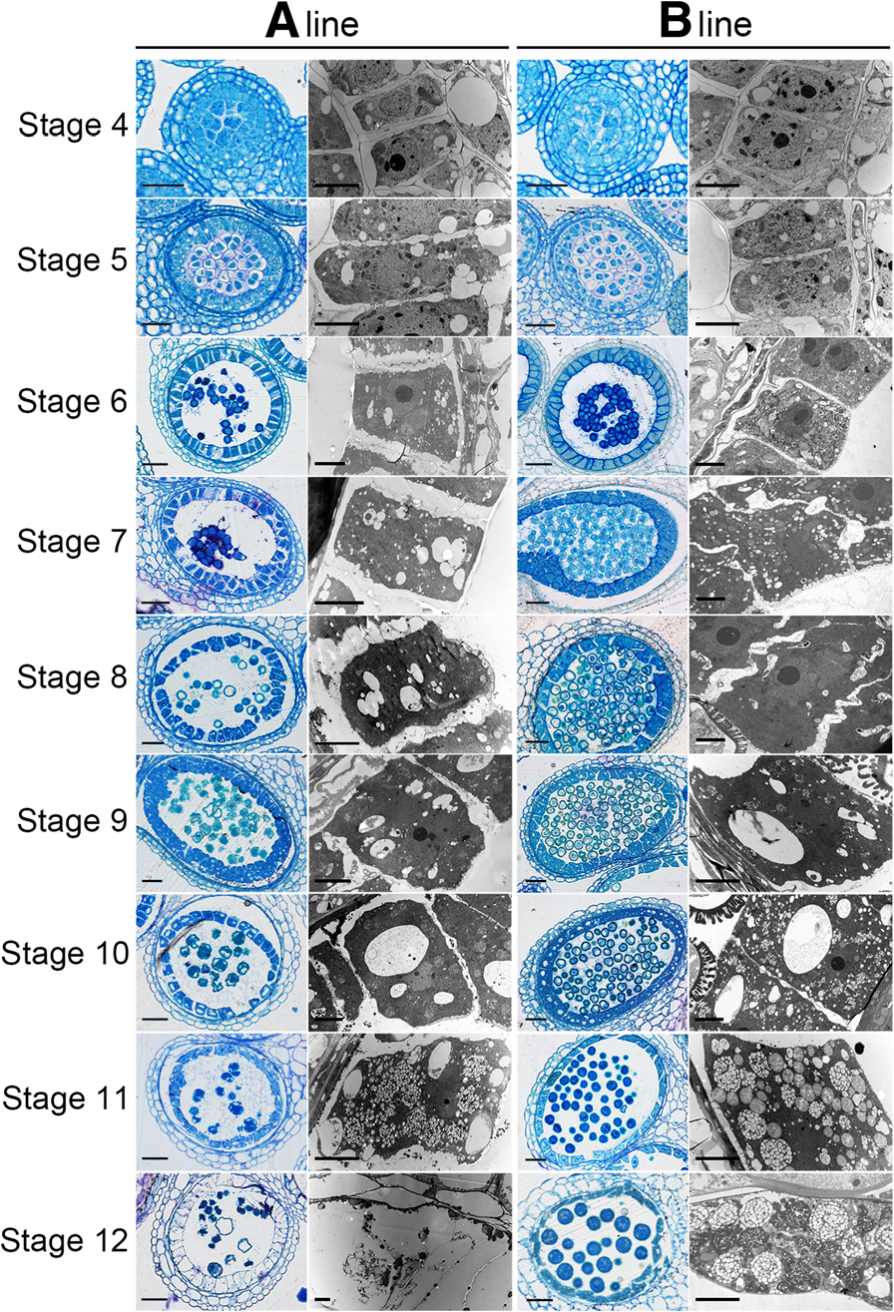
Analysis of tapetum development in the sterile line ‘Bcajh97-01A’ (A line) and the fertile line ‘Bcajh97-01B’ (B line) of *Brassica rapa* through semi-thin section and transmission electron microscopy observation. Stage 4, mother cell at pachytene/diplotene stage. Stage 5, mother cell at meiosis telophase I. Stage 6, tetrad stage. Stage 7, early uninucleate microspore stage. Stage 8, middle uninucleate stage. Stage 9, late uninucleate stage. Stage 10, early binucleate stage. Stage 11, middle binucleate stage. Stage 12, late binucleate stage. Scale bars in semi-thin sections of anthers: 50 μm. Scale bars in transmission electron microscopy observation: 4 μm

An in-depth cytological study and comparison was carried out on tapetum development of the sterile line utilizing semi-thin section and transmission electron microscopy (TEM) observation. No obvious difference was observed prior to tetrad stage. After meiosis, the sterile anther exhibited premature tapetal programmed cell death (PCD). The tapetal cells had indistinct cytoplasm and were loosely arranged. While, the tapetal cells of the fertile anther were tightly arranged and appeared metabolically active with abundant organelles. It was not until middle uninucleate stage, the tapetal cells of the fertile anther began to undergo PCD. At the binucleate stage, the tapetal cell in the fertile anther contained differentiating tapetosomes and elaioplasts, and the tapetum began to be thin. But in the sterile anther, no typical tapetosome was found. Elaioplasts with vague outlines differed slightly from the distinct elaioplast globules of the fertile anther which contained numerous round globuli within their stroma. At the late binucleate stage, most space between tapetal cells was consumed by elaioplasts and tapetosomes in the fertile anther, while the tapetal cytoplasm had been completely degraded in the sterile anther. However, the tapetal cell wall retained in the anther locule of the sterile line until the mature pollen stage when the whole tapetum layer was absent from the locule in the fertile anther (Fig. 3).

### Transcriptome assembly and annotation of Unigenes

To understand the pollen abortion-causing mechanism in ‘Bcajh97-01A/B’ and identify candidate genes contributing to anther and pollen development in *B. rapa*, via RNA-seq, we performed a detailed gene expression profiling on both of the sterile and fertile floral buds at five typical pollen developmental stages. After filtering out low quality data in both fertile and sterile libraries of each stage, over 14,500,000 reads (designated herein as “clean” reads) were remained. All clean reads were assembled by running Trinity, and 25,509,284 contigs (including 101,292 contigs > 200 bp) were generated. The contig length distribution was shown in Additional file 1: Figure S1. After clustering, 207,932 transcripts and 72,168 Unigenes were obtained (Additional file 2: Table S1), and 15,353 Unigenes (21.27%) were greater than 1000 bp in length and no Unigenes were shorter than 200 bp (Additional file 3: Figure S2).

For annotation, 72,168 Unigenes were subjected to BLASTX searches against the sequences in the NCBI non-redundant protein sequences (NR), Swiss-Prot, Gene Ontology (GO), the Clusters of Orthologous Groups (COG) and Kyoto Encyclopedia of Genes and Genomes (KEGG) databases. As a result, a total of 45,530 Unigenes (63.09% of all Unigenes) provided a significant BLAST result (Additional file 2: Table S2). Among the 45,530 Unigenes, approximately 32.83% could be annotated in COG classification. 14,946 Unigenes were classified into 24 function classifications (Additional file 4: Figure S3), which means that the identified genes are involved in various biological processes. GO classifications were also obtained to investigate the function of the Unigenes. In total, 31,340 annotated Unigenes were further classified into 49 functional groups (Additional file 5: Figure S4).

### Transcripts differentially expressed in the fertile and sterile floral buds

**Fig. 4:**
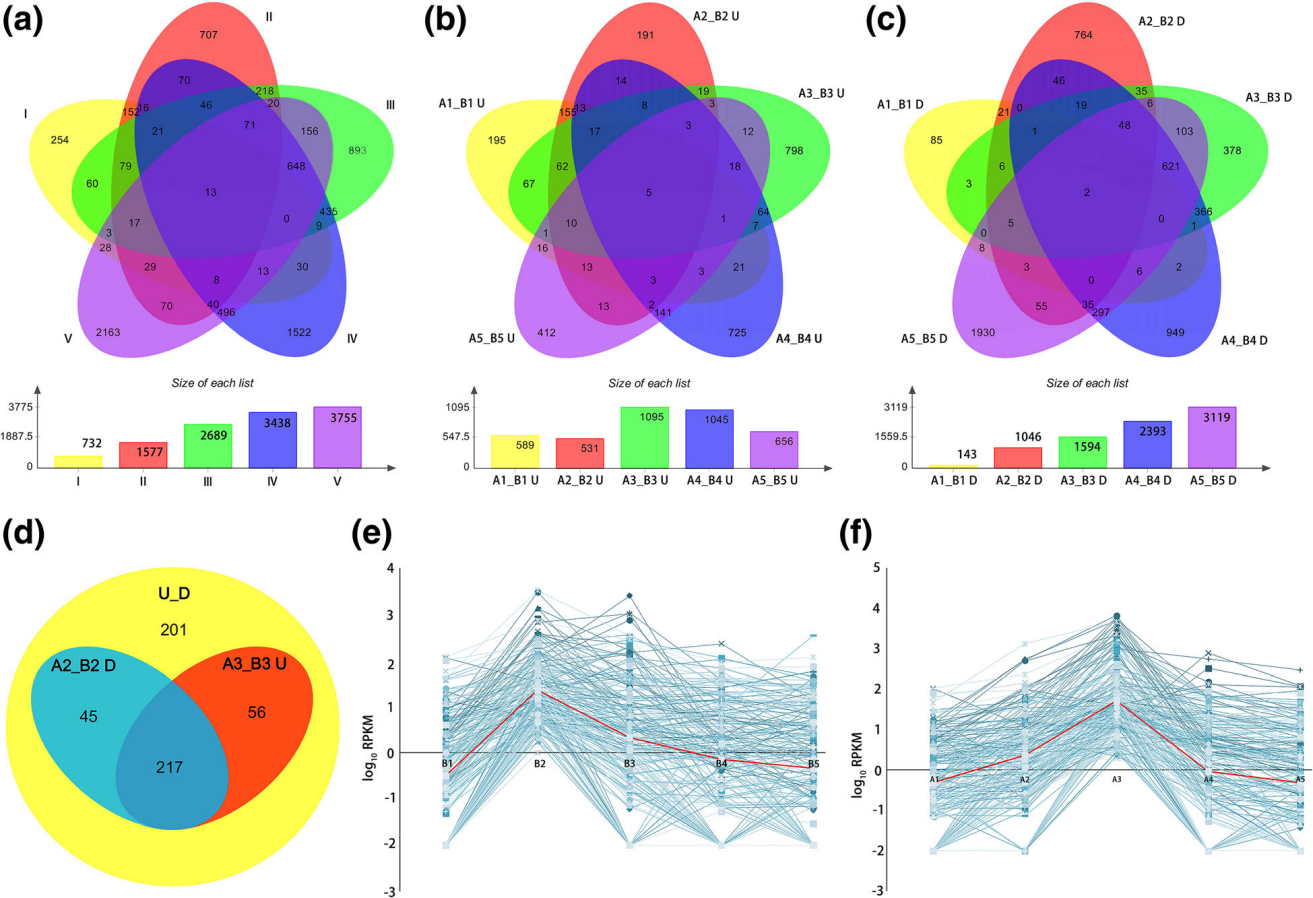
Venn diagrams and line charts showing differentially expressed genes (DEGs) between the sterile line ‘Bcajh97-01A’ and the fertile line ‘Bcajh97-01B’. **a** A summary of the numbers of DEGs at five developmental stages (I-V). **b** A summary of the numbers of DEGs up-regulated (U) in the sterile floral buds. **c** A summary of the numbers of DEGs down-regulated (D) in the sterile floral buds. **d** A summary of the numbers of DEGs showing opposite trend (U_D) at different developmental stages. **e** The expression (RPKM value) of 217 DEGs in the fertile floral buds at five developmental stages (B1-B5). Log_10_ (RPKM) equal to − 2 represents no expression. The red line represents the average expression. **f** The expression (RPKM value) of 217 DEGs in the sterile floral buds at five developmental stages (A1-A5). Log_10_ (RPKM) equal to − 2 represents no expression. The red line represents the average expression

With the restrictive conditions of False Discovery Rate (FDR) < 0.01 and log2 ratio > = 1.0, Unigenes that were differentially expressed in the fertile and sterile floral buds at five stages were identified. In total, 8288 genes (11.48% of Unigenes) were differentially expressed in at least one stage of the sterile floral buds compared with the fertile ones, and these genes were designated herein as DEGs (Fig. 4a and Additional file 2: Table S3). Among these DEGs, down-regulated genes accounted for the majority in each floral buds except A1 floral buds, i.e. the sterile floral buds at stage I (Fig. 4b and c). And as growth progresses, the number of down-regulated genes increased dramatically (Fig. 4c). Of the down-regulated genes, 4503 (77.70%) genes showed the lowest expression in stage IV or stage V in the sterile floral buds, the pollen maturation stage, reflecting the lack of mature pollen grains in the sterile line. More remarkable, 73.04% of down-regulated DEGs (764 genes) in stage II showed their differential expression only at this stage, suggesting that many genes are specific for meiosis or tetrad formation (Fig. 4c). In contrast, the number of the up-regulated genes changed gently, and 2030 (67.40%) genes were up regulated in the sterile floral buds at stage III or stage IV (Fig. 4b). Among the 8288 DEGs, 519 genes showed opposite trend at different stages (Fig. 4d). Hereinto, 217 genes were down-regulated at stage II, while up-regulated at stage III in the sterile floral buds compared with fertile ones. Further analysis showed that, as the fertile floral buds growth, these genes were down-regulated from stage II to stage III (Fig. 4e), however, the expression reached peak at stage III in the sterile floral buds (Fig. 4f). This result demonstrated that the expression of these genes were delayed during the developmental process of the sterile floral buds.

**Fig. 5:**
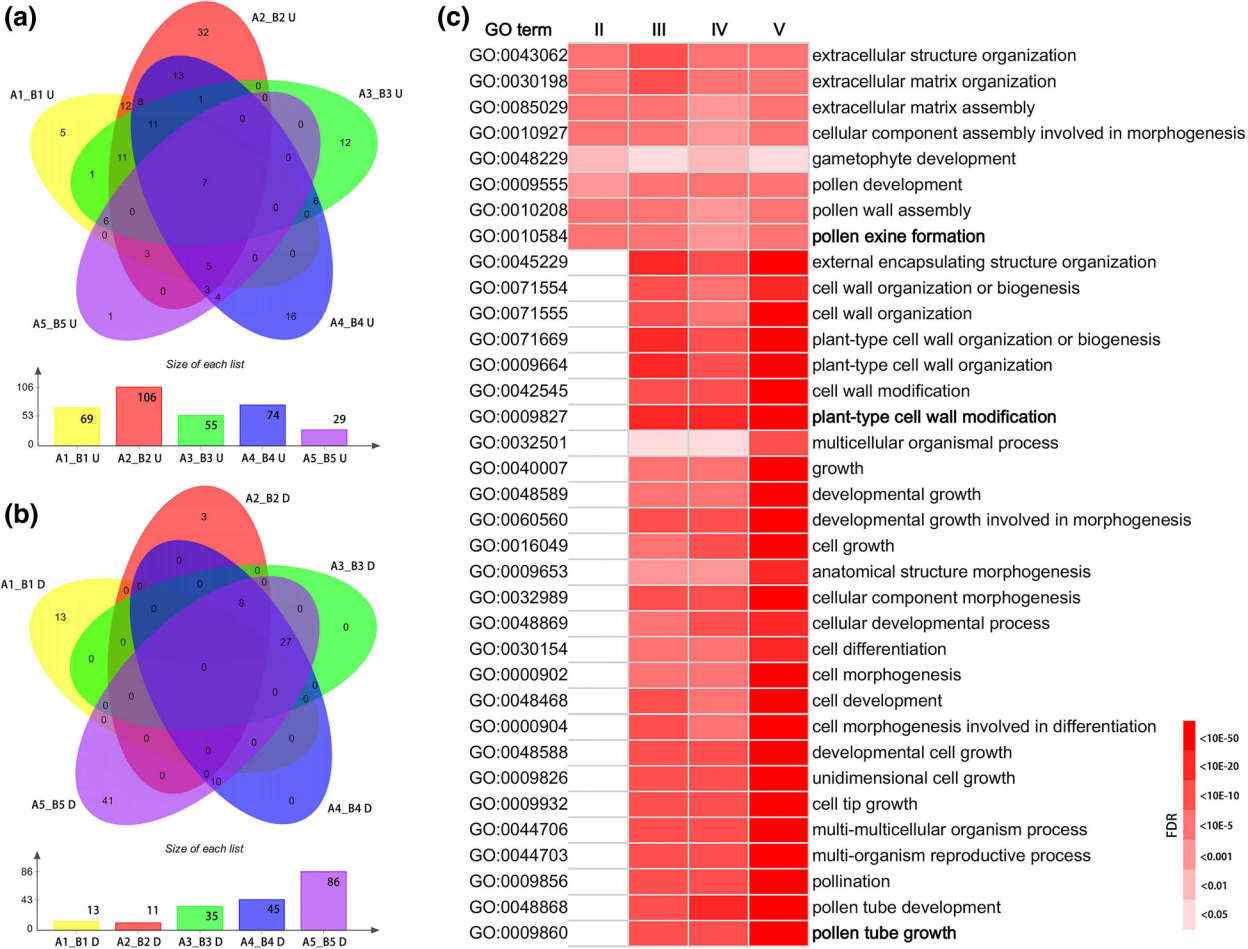
The numbers of significantly enriched GO terms by DEGs between the sterile line ‘Bcajh97-01A’ and the fertile line ‘Bcajh97-01B’ and some common Biological Processes GO annotation in some developmental stages.a.The numbers of significantly enriched Biological Processes GO terms by DEGs up-regulated (U) in the sterile floral buds. b. The numbers of significantly enriched Biological Processes GO terms by DEGs down-regulated (D) in the sterile floral buds. c. Eight and 27 Biological Processes GO terms were all significantly enriched by DEGs down-regulated in the sterile floral buds at stage II to V and stage III to V

**Fig. 6:**
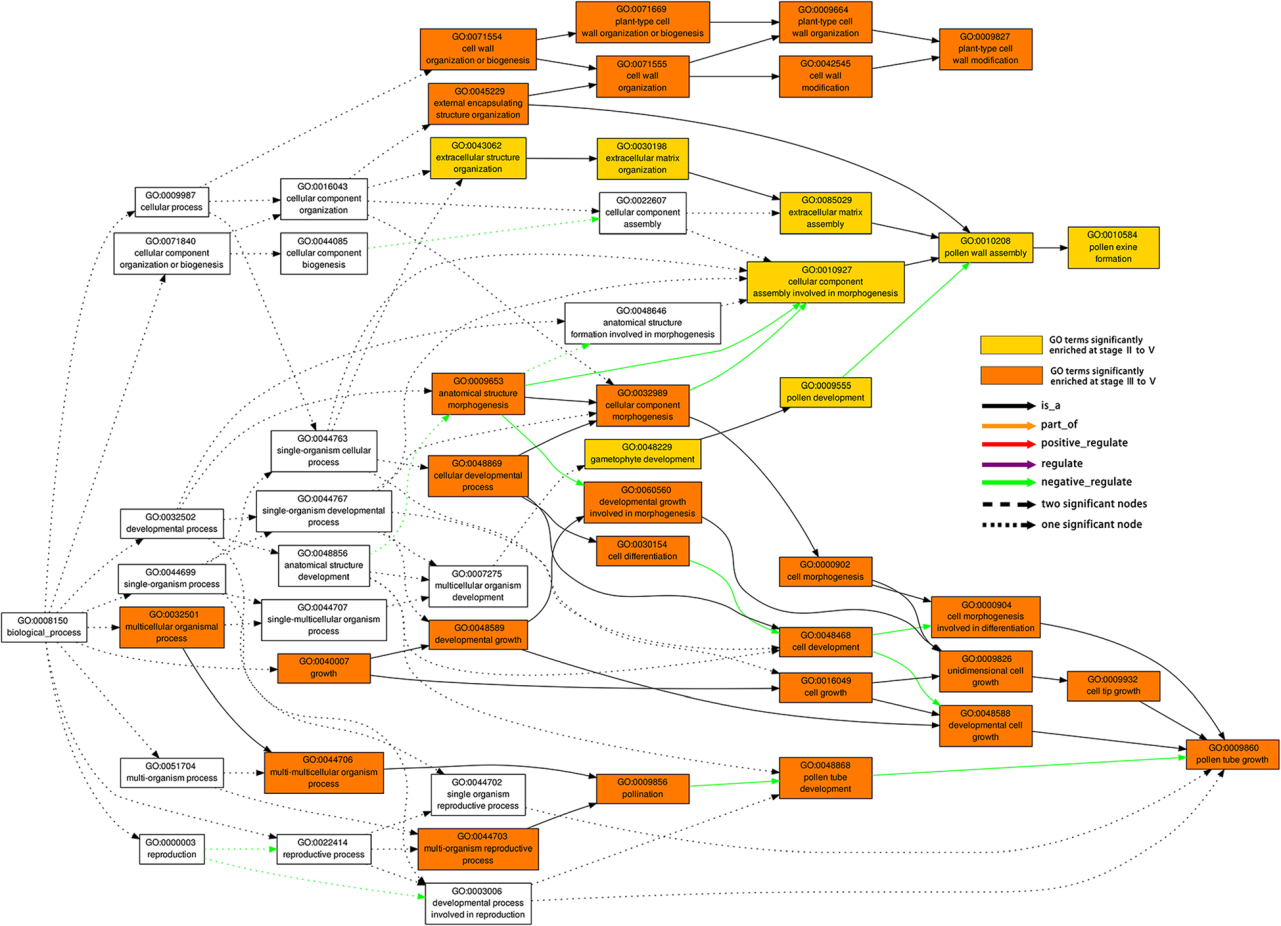
Some Biological Processes GO terms of DEGs with down-regulated levels in sterile buds at stage II to V. The color in each cell indicates FDR of the GO enrichment according to the scale shown, and blank cells indicate not significant

GO annotation information of the DEGs was selected for further functional analysis. To reveal significantly enriched GO terms in DEGs comparing to the transcriptome generated by this study, GO enrichment analysis of functional significance was performed using AgriGO, and the GO term with FDR ≤ 0.05 was defined as significantly DEGs enriched GO term. This analysis allowed us to identify the major biological processes (BP), molecular functions (MF) and cellular components (CC) with which DEGs were involved at each stage. For enriched BP, more GO terms were significantly enriched by the development of anther in down-regulated DEGs. At each stage, 13, 11, 35, 45 and 86 terms were revealed, respectively (Fig. 5b and Additional file 2: Table S4), which means that more biological processes were disturbed through the development. Several aspects are worthy of note. First, pollen development, pollen wall assembly, and pollen exine formation were GO terms significantly enriched at stage II through to stage V. Second, some GO terms related to cell wall organization or biogenesis were enriched at all stage III, IV and V. Third, a large number of GO terms finally pointing to pollen tube growth, such as pollination, cell differentiation, cell morphogenesis involved in differentiation, and cell growth, were also enriched at stage III through to stage V (Fig. 5c, Fig. 6 and Additional file 2: Table S4). In addition, pollen sperm cell differentiation were also enriched at stage IV and stage V (Additional file 2: Table S4). While at stage I, the enriched GO terms were different from the later four stages (Additional file 2: Table S4). For up-regulated DEGs, 69, 106, 55, 74 and 29 GO terms were significantly enriched at the five stages, respectively (Fig. 5a and Additional file 2: Table S4). Most of these GO terms were associated with responding to stimulus, especially in the earlier four stages. There were some enriched GO terms which were related to protein folding, morphogenesis, and cell death. It is worth mentioning that at stage II, the significantly enrichment GO terms involved programmed cell death, host programmed cell death induced by symbiont and toxin metabolic process (Additional file 2: Table S4), indicating that these DEGs may relate to the phenotype of premature tapetum degradation in the sterile line.

For enriched MF, it is worth pointing out that one of hydrolase activity, polygalacturonase activity were remarkably enriched at stage III, IV, and V (Additional file 2: Table S4), with the gene number increasing through development. Another enriched MF and CC GO terms were also showed in Additional file 2: Table S4. Using the KEGG database, the complex biological behaviors of genes were further studied. A total of 56, 83, 100, 99 and 103 biological pathways were identified by KEGG pathway analysis for the DEGs at five stages, and pathway enrichment analysis showed that there were 11, 11, 12, 11 and 18 pathways were significantly enriched, respectively (Additional file 2: Table S5). There were 10, 8.16, 8.36, 9.02 and 5.28% DEGs belonging to the enriched pathway “plant hormone signal transduction” at the five stages, respectively. These DEGs were involved in complicated regulatory network of plant hormone signal transduction, including auxin, cytokinine, abscisic acid, ethylene, brassinosteroid, jasmonic acid and salicylic acid. While, gibberellin signal transduction was not included. “Starch and sucrose metabolism” was significantly enriched at the later four stages. According to the KEGG maps, the up- or down-regulated DEGs belonging to this pathway mainly took part in the regulation of saccharometabolism, such as the biosynthesis of pectin, the production of monosaccharide and the degradation of pectin or cellulose, which suggests that early aberrant meiotic cytokinesis may finally affects the pollen wall modification.

### Callose metabolism-related genes showed dramatically altered expression in the sterile line

Callose, a polymer of β-1, 3 glucans, serves as a temporary wall to separate newly formed microspores in the tetrad and acts as the mold for primexine. Callose defects can affect pollen wall formation and pollen viability. The expression changes of 15 callose synthase genes were analyzed in the sterile line. None of these 15 genes displayed changed expression at stage II, suggesting normal synthesis of callose required for tetrad formation.

The accurate timing of callase activation is critical for normal callose degradation. Therefore, we analyzed the expression of all *B. rapa* genes encoding putative β-1, 3-glucanases. Among the 52 genes that were detected in the sequencing, 13 genes showed different expression between the fertile and sterile lines at least one floral developmental stage. Two major groups were distinguished according to the dominant expression pattern of these genes in the fertile line. One group includes seven early-expressed genes, and the other one contains six genes that expressed during the late pollen maturation stages. All of the six late-expressed genes showed reduced expression levels in the sterile floral buds. Interestingly, seven early-expressed genes showed consistent changes in gene expression between the fertile and sterile lines with a decrease first at stage II followed by a remarkable up-regulation at stage III in the sterile floral buds. For example, the expression levels of Bra032758 and Bra037057, two orthologs of Arabidopsis *A6*, were nearly reduced by half first but then up-regulated over 30-fold, and 60-fold respectively. Bra001918 showed a sharp decrease (< 5% remained) at stage II followed by an over 70-fold increase at stage III. While, further analysis of these genes revealed that, in the fertile floral buds, they were almost expressed specifically at one developmental stage, which is stage II. As regards for regulation on callose metabolism, Bra004288, the ortholog of Arabidopsis *CDM1* was expressed at stage II specifically in the fertile floral buds and decreased dramatically in the sterile floral buds (Table 1).

**Table 1:** Callose metabolism-related genes showed dramaticaly altered expression

*Brassica rapa*						Arabidopsis		
Locus	B1/A1	B2/A2	B3/A3	B4/A4	B5/A5	Locus	Gene name	Description
Bra037213	–	–	4.9	5.6	9.4	AT2G13680	*GSL2*	Callose synthase
Bra004288	–	1.8	–	–	–	AT1G68200	*CDM1*	Zinc finger C-×8-C-×5-C-×3-H type family protein
Bra026644	–	–	–	–	1.8	AT2G01630	*–*	Glycosyl hydrolases family 17 protein
Bra036718	–	–	–	2.2	–	AT1G64760	*–*	Glycosyl hydrolases family 17 protein
Bra010330	–	–	–	–	2.0	AT4G29360	*–*	Glycosyl hydrolases family 17 protein
Bra028988	–	–	–	2.4	–	AT5G55180	*–*	Glycosyl hydrolases family 17 protein
Bra037795	–	–	7.0	7.1	8.7	AT5G64790	*–*	Glycosyl hydrolases family 17 protein
Bra031901	–	–	–	–	6.2	AT5G64790	*–*	Glycosyl hydrolases family 17 protein
Bra003475	–	1.1	–	2.9	–	AT3G61810	*–*	Glycosyl hydrolases family 17 protein
Bra001918	−2.7	5.3	−6.2	–	–	AT3G23770	*–*	Glycosyl hydrolases family 17 protein
Bra019084	–	4.4	−4.5	–	2.3	AT4G26830	*–*	Glycosyl hydrolases family 17 protein
Bra037057	–	–	−6.0	–	–	AT4G14080	*A6*	Glycosyl hydrolases family 17 protein
Bra032758	−2.1	1.2	−5.1	1.8	–	AT4G14080	*A6*	Glycosyl hydrolases family 17 protein
Bra020110	–	–	−7.1	–	–	AT5G20330	*–*	Glycosyl hydrolases family 17 protein
Bra014979	–	–	−4.3	–	–	AT3G23770	*–*	Glycosyl hydrolases family 17 protein

All values are expressed in terms of log2FC (fold change), so that positive values indicate depression of gene expression in male sterile line ‘Bcajh97-01A’. Shot dashes represent either no significant difference or no expression

### The expression of genes presumed to be involved in the formation of pollen exine was changed in the sterile line

GO analysis revealed that GO term “pollen exine formation” was enriched at all the late three developmental stages and “sporopollenin biosynthetic process” was specially enriched at stage III, suggesting seriously impaired pollen exine formation. The Brassica and Arabidopsis genera share about 85% exon sequence similarity [35], and since genes regulating anther and pollen development have been well established in Arabidopsis by genetic and molecular biological studies, here, we analyzed the expression of *B. rapa* orthologs of Arabidopsis genes that are known to be involved in the formation of pollen exine. It was known that the biosynthesis and transport of the lipidic and phenolic precursors was important for the formation of pollen exine [8]. Therefore, we examined the expression of *B. rapa* orthologs of Arabidopsis genes that were associated with lipid metabolism and phenolic metabolism during pollen wall development. Our results indicated that all the *B. rapa* orthologs of those tapetum-specific genes including *MALE STERILITY 2* (*MS2*), *CYP703A2*, *CYP704B1*, *ACYL-COA SYNTHASE 5* (*ACOS5*), *POLYKETIDE SYNTHASE A* (*PKSA*), *POLYKETIDE SYNTHASE B* (*PKSB*) and *TETRAKETIDE α-PYRONE REDUCTASE 2* (*TKPR2*) were dramatically affected in the sterile floral buds. It was worth mentioning that they showed a decrease in expression at stage II and then a significant up-regulation at stage III in the sterile floral buds, compared with the fertile ones.

Furthermore, our analyses revealed that six orthologous genes related to transport of precursors required for exine development showed different expression levels between the sterile and fertile lines. Among them, Bra039378 and Bra005048, the orthologs of Arabidopsis *ATP-BINDING CASSETTE G26* (*ABCG26*) and *ABCG1*, performed with a similar change in expression as with those lipid and phenolic metabolism-related genes mentioned above. And other four orthologs of Arabidopsis ABCG transporters were all down-regulated in the sterile line. However, four orthologs of Arabidopsis lipid transfer protein genes exhibited increased expression levels ranging from 3.71-fold to 537.30-fold in the sterile floral buds at the late pollen developmental stages. The above data suggested that transport of precursor across anther tissue during pollen development was partially affected in the sterile floral buds.

We also examined the expression of several orthologs of Arabidopsis transcription factors (TFs) which had been reported to form regulatory networks to control pollen wall development. The expression of *BrDYT1* (Bra013519) was up-regulated at stage II in the sterile floral buds while some genes essential for post-meiotic tapetal function including *BrAMS* (Bra002004 and Bra013041), *BrMYB80* (Bra002847 and Bra035604) and *BrMS1* (Bra002401) decreased first in expression at stage II and then increased at stage III. In addition, Bra004689, the ortholog of Arabidopsis *bZIP34* exhibited reduced expression level in the late-stage sterile floral buds (Table 2).

**Table 2:** Pollen exine formation-related genes showed changed expression

Classification	*Brassica rapa*						Arabidopsis		
	Locus	B1/A1	B2/A2	B3/A3	B4/A4	B5/A5	Locus	Gene name	Gene expression pattern
Lipid metabolism	Bra038691	− 2.7	4.0	− 2.8	–	–	AT3G11980	*MS2*	tapetum shortly after release of microspore from tetrad
	Bra033272	−2.6	3.6	−6.1	–	5.6	AT1G01280	*CYP703A2*	tapetum and microspore specific
	Bra004386	−1.4	1.7	−4.0	–	–	AT1G69500	*CYP704B1*	anther-specific
Phenolic metabolism	Bra036646	−2.4	1.4	−8.5	–	–	AT1G62940	*ACOS5*	tapetum-specific
	Bra011566	−1.7	2.5	−8.1	–	–	AT4G34850	*PKSA*	tapetum-specific
	Bra017147	−1.7	3.3	−6.5	–	–	AT1G02050	*PKSB*	tapetum-specific
	Bra004316	−3.0	–	−4.4	–	–	AT1G68540	*TKPR2*	tapetum-specific
Transporters	Bra039378	–	1.5	−3.2	–	–	AT3G13220	*ABCG26*	tapetum-specific
	Bra005048	–	1.4	−5.2	–	–	AT2G39350	*ABCG1*	tapetum-specific
	Bra026352	–	–	2.3	–	–	AT4G27420	*ABCG9*	tapetum-specific
	Bra021598	–	–	4.2	4.6	4.7	AT2G29940	*ABCG31*	tapetum-specific
	Bra000469	–	1.9	3.9	3.3	3.3	AT2G29940	*ABCG31*	tapetum-specific
	Bra014776	–	3.2	3.5	2.0	2.7	AT3G55090	*ABCG16*	tapetum-specific
	Bra038907	−1.8	–	–	–	−8.9	At3G51590	*LTP12*	anther wall tapetum
	Bra028294	−2.8	–	−6.8	–	–	AT5G52160	*–*	flower stage 9
	Bra009282	–	–	−2.1	–	–	AT5G07230	*–*	flower stage 9,10,11
	Bra012819	−3.9	–	−8.2	–	–	AT3G52130	*–*	flower stage 9
Transcription factors	Bra013519	–	−3.0	4.3	5.3	–	AT4G21330	*DYT1*	highly expressed in the tapetum, meiocytes and microspores
	Bra002004	–	–	−1.4	1.9		AT2G16910	*AMS*	tapetum
	Bra013041	–	–	−1.2	–	–	AT2G16910	*AMS*	tapetum
	Bra002847	–	3.8	−6.1	–	–	AT5G56110	*MYB80*	tapetum, middle layers and developing microspores
	Bra035604	–	2.2	−8.9	–	–	AT5G56110	*MYB80*	tapetum, middle layers and developing microspores
	Bra002401	–	5.3	−3.7	–	–	AT5G22260	*MS1*	tapetum shortly after microspore release from tetrad
	Bra004689	–	–	1.7	–	–	AT2G42380	*bZIP34*	anthers and pistils

All values are expressed in terms of log2FC (fold change), so that positive values indicate depression of gene expression in male sterile line ‘Bcajh97-01A’. Shot dashes represent either no significant difference or no expression

### Screening of potential tapetum development and function-related genes

As the innermost sporophytic cell encasing the developing pollen, tapetum plays an essential role controlling pollen exine formation and pollen development. Previous expression analysis of DEGs has revealed that a part of tapetum development and function-related genes displayed a similar developmental expression change. As genes associated with the same metabolic pathway are perceived to be more highly coexpressed than genes from different pathways, here, we analyzed those genes which were down-regulated at stage II and then up-regulated at stage III in the sterile line to screen potential participants in tapetum development and function. The information of their homologous genes in Arabidopsis provided deeper understanding of functions these genes may perform during anther development.

Besides those pollen exine formation-involved genes we have mentioned above, twenty-five other genes encoding different kinds of proteins drew our attention. Their homologous genes in Arabidopsis were found to be specifically expressed or highly expressed at flower stage 9 according to the data of Arabidopsis eFP browser (http://bar.utoronto.ca/efp/cgi-bin/efpWeb.cgi), coinciding with those of tapetum development and function-related genes that have been reported to be involved in pollen exine formation. What’s more, nearly all these genes showed variable expressional changes in several Arabidopsis floral mutants (Table 3). According to the microarray data, those genes with expressional changes in *ems1*, *spl*, *tdf1* and *ams* were all down-regulated without exception. Interestingly, nearly two-thirds of those genes with expressional changes in *ms1* exhibited up-regulated expression coinciding with expression analysis at stage III in our study. Further analysis revealed that Arabidopsis homologs of eight genes, Bra038803, Bra020920, Bra028324, Bra032758, Bra029151, Bra022571, Bra028286 and Bra016531, displayed absolutely similar expressional changes to those function-known tapetum-related genes during distinct flower developmental stages in the *ms1* mutant.

**Table 3:** Tapetum development and function-related genes

Classification	*Brassica rapa* Locus	Arabidopsis Locus	Description	Arabidopsis microarray data				
				WT/*ems1*^a^	WT/*spl1*^a^	WT/*tdf1*^b^	WT/*ams*^c^	WT/*ms1*^d^
Known genes	Bra002847	AT5G56110	AtMYB80	2.2	2.5	–	–	−2.2
	Bra035604	AT5G56110						
	Bra002401	AT5G22260	MALE STERILITY 1	–	–	–	–	−2.7
	Bra039378	AT3G13220	ABCG26	52.2	56.7	2.2	5.9	−1.6
	Bra002004	AT2G16910	AMS	31.8	28.8	3.3	–	−2.1
	Bra013041	AT2G16910						
	Bra033272	AT1G01280	CYP703	47.9	43.6	12.0	9.9	−2.9
	Bra004386	AT1G69500	CYP704B1	117.5	127.8	8.6	11.6	−2.7
	Bra038691	AT3G11980	MALE STERILITY 2	42.8	50.8	31.7	17.5	−2.2
	Bra017147	AT1G02050	LESS ADHESIVE POLLEN 6	17.4	14.8	6.2	6.5	–
	Bra011566	AT4G34850	LESS ADHESIVE POLLEN 5	–	–	22.4	8.7	–
	Bra036646	AT1G62940	ACYL-COA SYNTHETASE 5	53.5	93.4	18.4	6.9	−4.0
Taptum specific genes	Bra038803	AT4G20420	tapetum specific protein TAP35/TAP44	33.9	53.2	11.7	–	−7.2
	Bra020920	AT4G20420						
	Bra028324	AT3G42960	ATA1/TAPETUM 1	61.4	106.6	2.9	8.2	−1.6
Carbonhydrate metabolism and transport	Bra020859	AT4G22080	pectin lyase-like superfamily protein	–	7.5	–	–	3.4
	Bra013442	AT4G20050	QRT3	34.3	37.3	5.3	6.3	−1.5
	Bra023600	AT5G17200	pectin lyase-like superfamily protein	–	1.8	–	–	2.1
	Bra001918	AT3G23770	Glycosyl hydrolases family 17 protein	28.5	25.0	4.9	–	4.8
	Bra032758	AT4G14080	Glycosyl hydrolases family 17 protein	82.9	204.5	54.0	8.6	−3.9
	Bra022636	AT5G53190	nodulin MtN3 family protein	37.2	54.3	–	1.2	1.1
Oxidation reduction	Bra015740	AT1G76470	NAD(P)-binding Rossmann-fold superfamily protein	18.7	18.0	11.4	13.1	22.7
	Bra021337	AT3G57620	glyoxal oxidase-related protein	7.7	8.2	–	–	2.4
	Bra029151	AT5G51950	glucose-methanol-choline oxidoreductase family protein	12.2	14.8	–	3.6	−1.6
	Bra022571	AT5G51950						
	Bra028286	AT5G51950						
The others	Bra020777	AT3G06100	NOD26-LIKE INTRINSIC PROTEIN 7	8.5	12.2	7.5	5.4	−2.1
	Bra037505	AT5G48210	prolamin-like protein (DUF1278)	5.2	6.7	7.3	–	9.6
	Bra020712	AT5G48210						
	Bra015870	AT1G75050	pathogenesis-related thaumatin superfamily protein	3.8	6.1	–	4.6	1.3
	Bra032134	AT2G23945	eukaryotic aspartyl protease family protein	–	–	–	–	–
	Bra011161	AT4G30030	eukaryotic aspartyl protease family protein	–	–	–	–	–
	Bra009795	AT5G24820	eukaryotic aspartyl protease family protein	49.4	62.3	–	–	–
	Bra030647	AT1G06280	LOB DOMAIN-CONTAINING PROTEIN 2	–	18.4	19.5	–	8.2
	Bra018697	AT1G47980	desiccation-like protein	19.8	17.5	32.6	17.7	6.3
	Bra018660	AT1G08065	ALPHA CARBONIC ANHYDRASE 5	26.9	24.5	4.9	-	-
	Bra016531	AT1G18960	myb-like transcriptional regulator family protein	-	7.5	9.6	-	−4.2

The values in the column of “WT/*ms1*” are expressed in terms of log2FC (fold change) and the values in other columns are expressed in terms of the ration of wild type to mutant. Shot dashes represent either no significant difference or no expression.

^a^Genes showing expression change in *spl* and *ems1* mutant [36]

^b^Genes showing expression change in *tdf1* mutant [15]

^c^Genes showing expression change in *ams* mutant [16]

^d^Genes showing expression change in *ms1* mutant [37]

**Fig. 7:**
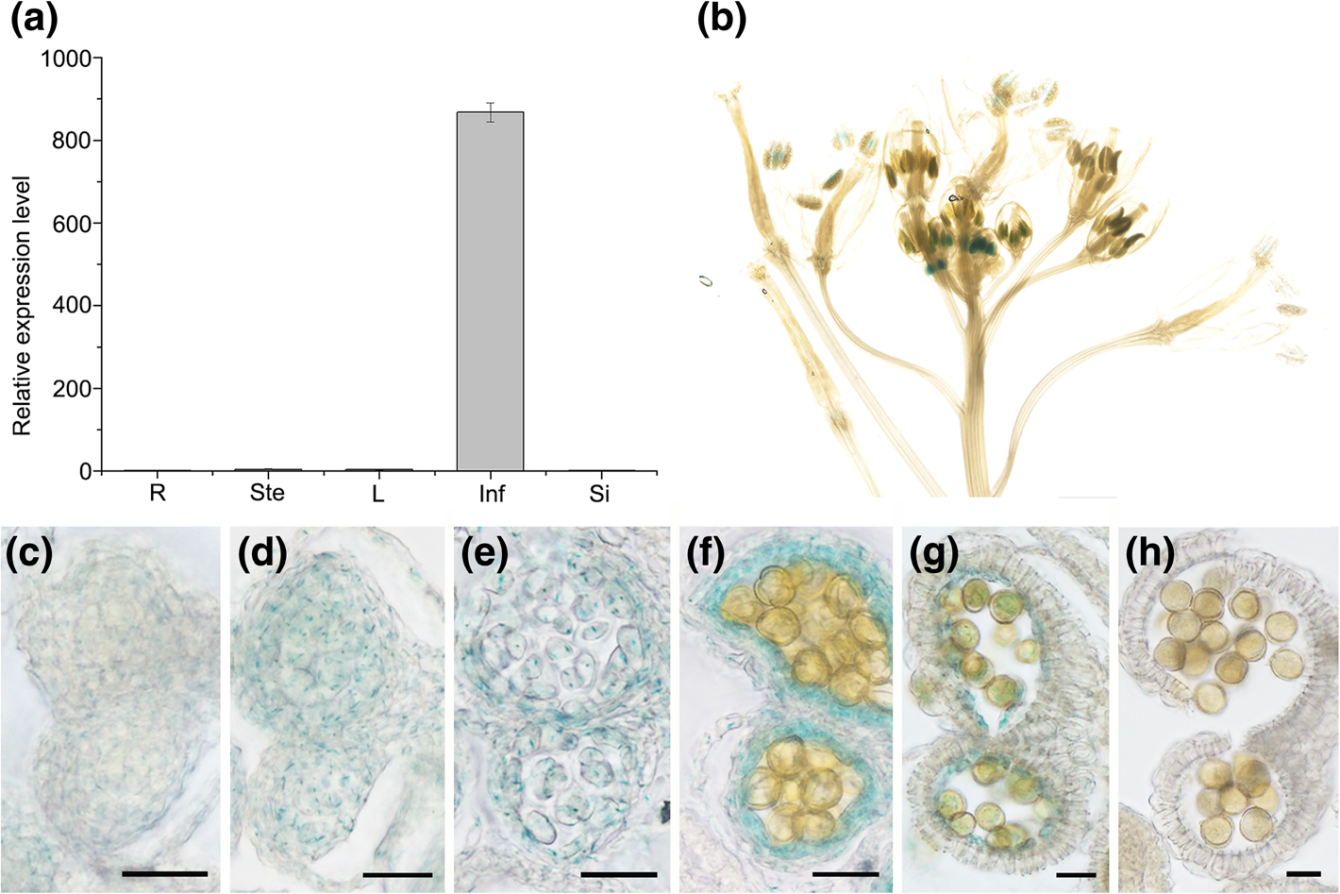
Expression pattern analysis of Bra016531. **a** Quantitative RT-PCR analysis of Bra016531 transcripts in different tissues of *Brassica rapa*: roots (R), stems (Ste), leaves (L), inflorescences (Inf) and siliques (Si). **b** Bra016531 promoter-GUS activity. (**c**-**h**) Thin sections of anthers from Bra016531pro::*GUS*-expressing plants. c.pollen mother cell stage. d.meiosis stage. e.tetrad stage. f.early vacuolated stage.g.tapetum degeneration stage. h.mature pollen stage. Scale bars, 20 μm

To verify the function of these newly identified genes that may be involved in tapetum development, we selected one of them, Bra016531, which encodes a single MYB domain-containing protein, to be further investigated. Quantitative RT-PCR analysis of Bra016531 transcripts in different tissues revealed that Bra016531 was specific to inflorescence (Fig. 7a). Its detailed expression pattern was then examined in Arabidopsis plants expressing a Bra016531 promoter-driven β-glucuronidase gene (Bra016531pro::*GUS*). In more than 10 independent transgenic lines, Bra016531 promoter-driven GUS activity was apparent only in the anthers of young flowers (Fig. 7b). Then, Bra016531 promoter-driven GUS expression was examined in anther cells using thin sections taken from floral buds at various developmental stages (Fig. 7c-h). Blue GUS signal was first detected both in sporocyte and tapetal cells during meiosis (Fig. 7d), and then in tetrads and tapetal cells (Fig. 7e). Later, the signal was detected mainly in tapetal cells at the early vacuolate stage (Fig. 7f). These temporal and spatial patterns of Bra016531 expression supports our hypothesis that Bra016531 is associated with tapetum development and function during pollen development.

### A high proportion of cell wall modification-related genes were down regulated in the sterile line

**Fig. 8:**
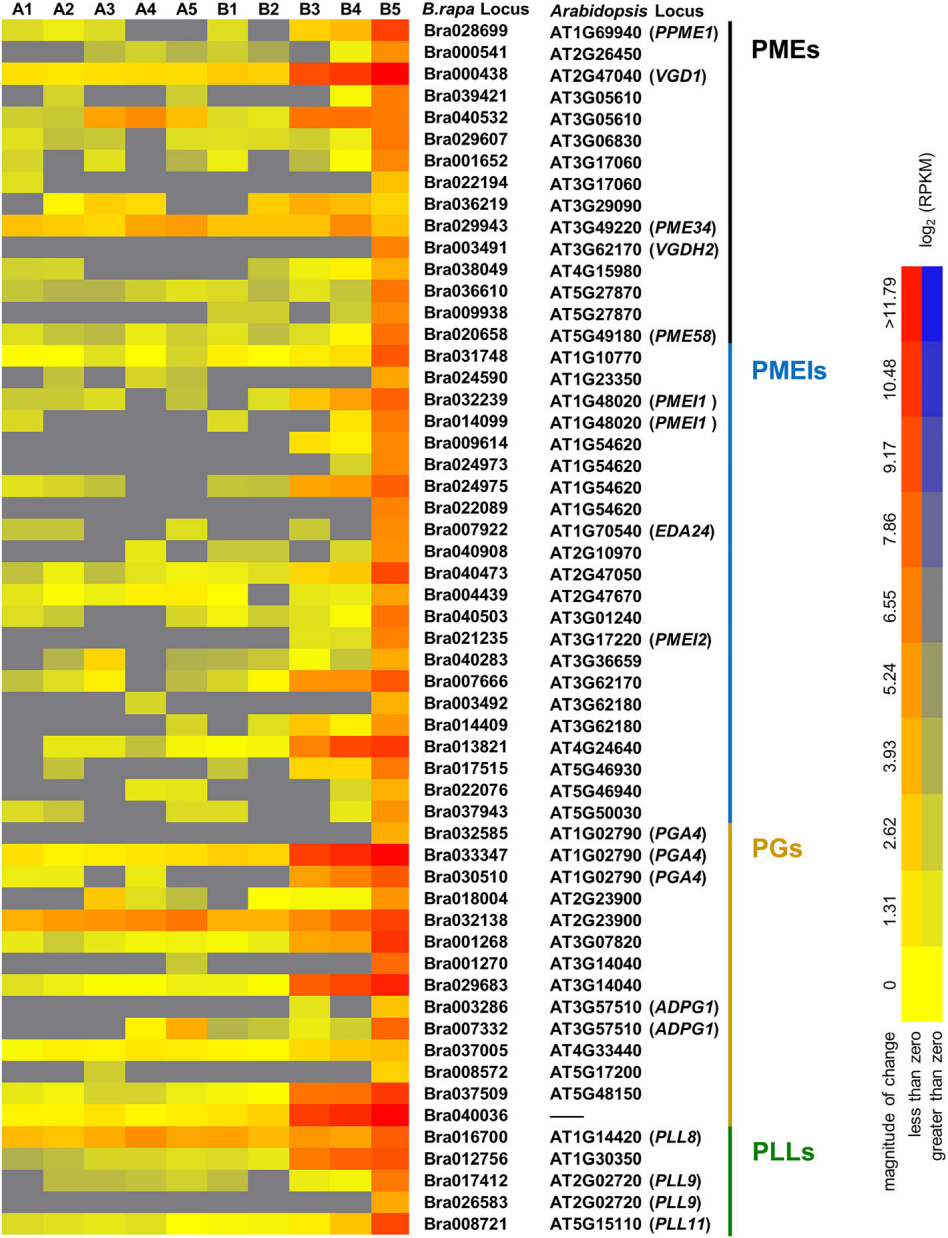
Hierarchical cluster display of the differentially expressed cell wall hydrolytic enzyme-coding genes in *Brassica rapa*. The color scale bar shown under the cluster indicates the maximum and minimum brightness values that represent the values of log_2_ (RPKM)

We found a high proportion of cell wall modification-related genes were down regulated in the sterile floral buds, especially at stage V with more than 12% of the total genes identified by GO analysis. Among these genes, some cell wall hydrolytic enzyme-encoding gene families that involved in polysaccharide metabolism captured our attention. Among PMEs family, 15 genes were down-regulated in the sterile floral buds. Most of these *PME*s had very low or undetectable expression during the early stages but went up dramatically at the mature pollen stage. For example, Bra000438 (homolog of *AtVGD1*), Bra003491 (homolog of *AtVGDH2*) and Bra028699 (homolog of *AtPPME1*) were highly and specifically expressed in the fertile floral buds at stage V. The similar general tendency was apparent in another cell wall modification-associated gene family which is closely related to PMEs, that is pectin methylesterase inhibitor protein (PMEI) gene family. Twenty-two *PMEI*s were expressed specifically in the fertile floral buds including counterparts of *AtPMEI1* (Bra014099 and Bra032239) and *AtPMEI2* (Bra021235). As for PGs family, most *PG*s had very low or undetectable expression during the early stages then went up dramatically at stage V. Among these 14 *PG*s, some genes showed extremely high expression at the last stage, such as Bra029683 and Bra033347. However, in the sterile floral buds, these *PG*s showed almost undetectable expression. In addition, five genes encoding PLLs, namely Bra012756, Bra017412, Bra017412, Bra008721 and Bra016700, expressed at an extremely high level at the mature pollen stage. And except for Bra016700, the other four genes presented a specific expression in the fertile floral buds (Fig. 8).

### Transcription factors showed expressional changes in the sterile line

According to the information of transcription factors in Brassica database (http://brassicadb.org/brad/) and Plant Transcription Factor Database (http://planttfdb.cbi.pku.edu.cn/) as well as annotations of Unigenes in transcriptome analysis, we identified 2567 Unigenes that encode putative transcription factors (TFs). These TFs were divided into 65 gene families. Different percentages of Unigenes in 53 gene families displayed changed expression patterns in the sterile floral buds compared with the fertile floral buds. Among those over-represented were the HSF family (38%), the LBD family (36%), the NAC family (33%), the MADS family (32%), the MYB-related family (28%), the AP2 family (23%), the C2H2 family (22%) and the BHLH family (21%). In contrast, the E2F (8%), Aflin (7%), GRF (6%) and FHA (5%) families were all under-represented. It’s worth mentioning that DEGs belonging to the NAC (48), BHLH (41), AP2 (40), MYB (32), MADS (32), C2H2 (27) and WRKY (26) families accounted for half of all 493 putative TFs that with changed expression patterns (Additional file 2: Table S6).

**Fig. 9:**
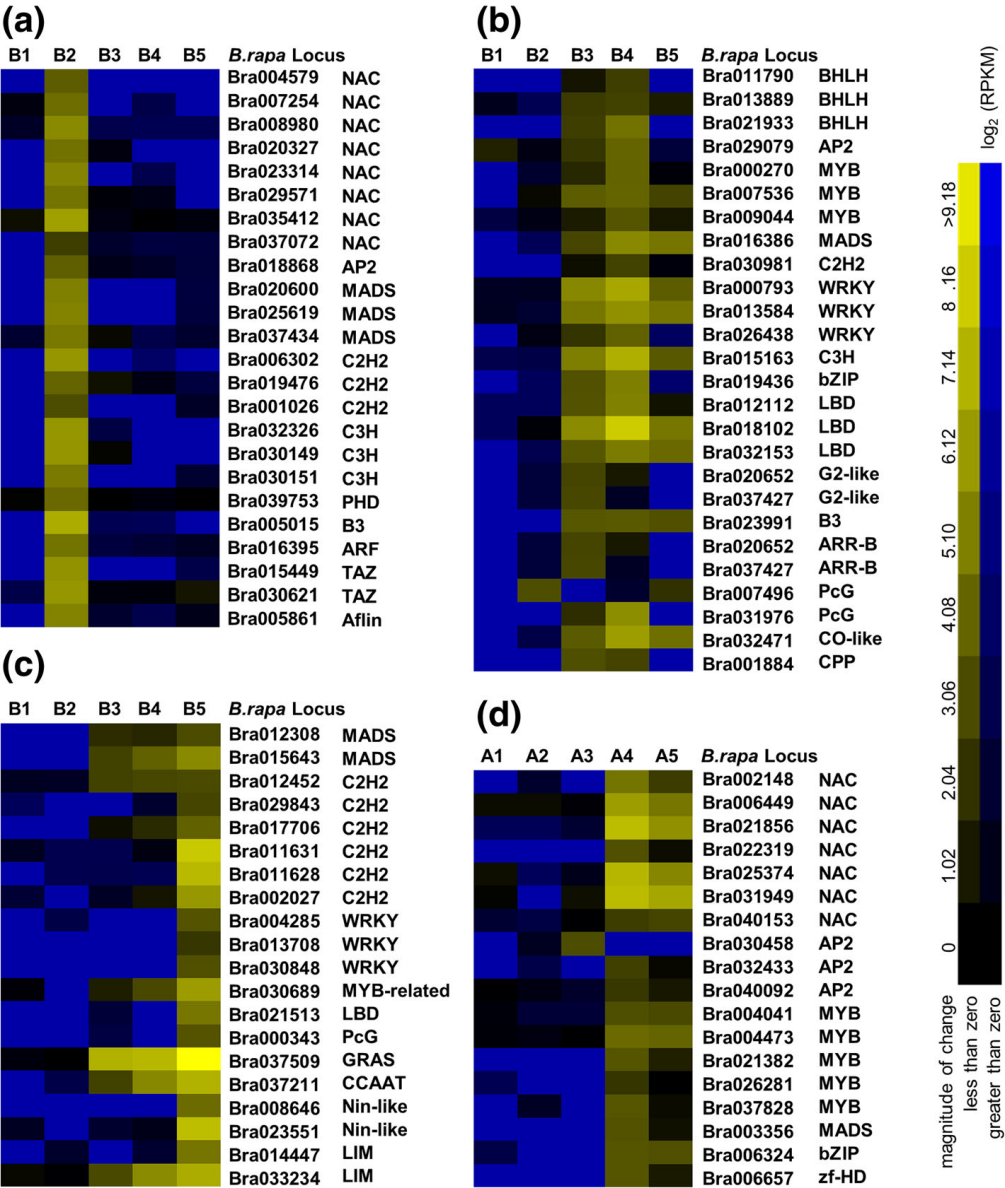
Expression of transcription factors (TFs) showing fertile and sterile floral buds-specific features throughout anther development. The intensities of the colors increase with increasing expression levels, as indicated at the bottom. **a** the fertile floral buds-specific TFs exclusively expressed at tetrad stage. **b** the fertile floral buds-specific TFs highly expressed at ninucleate stage and binucleate stage. **c** the fertile floral buds-specific TFs mainly expressed at mature pollen stage. **d** the sterile floral buds-specific TFs

Most of these differently expressed TFs were constitutively expressed in both of the sterile and fertile lines, but 70 genes were expressed specifically in the fertile floral buds, whereas 18 genes were specific to the sterile floral buds. Among those 70 genes, 24 genes were exclusively expressed at stage II and they belonged to the NAC, AP2, MADS, C2H2, C3H, PHD, ABI3, ARF, TAZ and Aflin families (Fig. 9a). It is noteworthy that NAC TFs accounted for one-thirds of these 24 genes revealed the important roles that NAC TFs play in regulating early anther development. We also found 20 genes including three orthologs of Arabidopsis *DAZ1*, *DAZ2* and *DAZ3*, exhibited remarkably high expression at mature pollen stage compared with the other four stages (Fig. 9c). Eighteen sterile floral buds-specific genes which belonged to the NAC, AP2, MYB, MADS, bZIP and zf-HD families were mainly expressed at stage IV and stage V, suggesting that they were up-regulated at later pollen development processes in the sterile line (Fig. 9d).

### Pollen development-related genes were covalently modified by H3K4me^3^ and H3K27me^3^

**Fig. 10:**
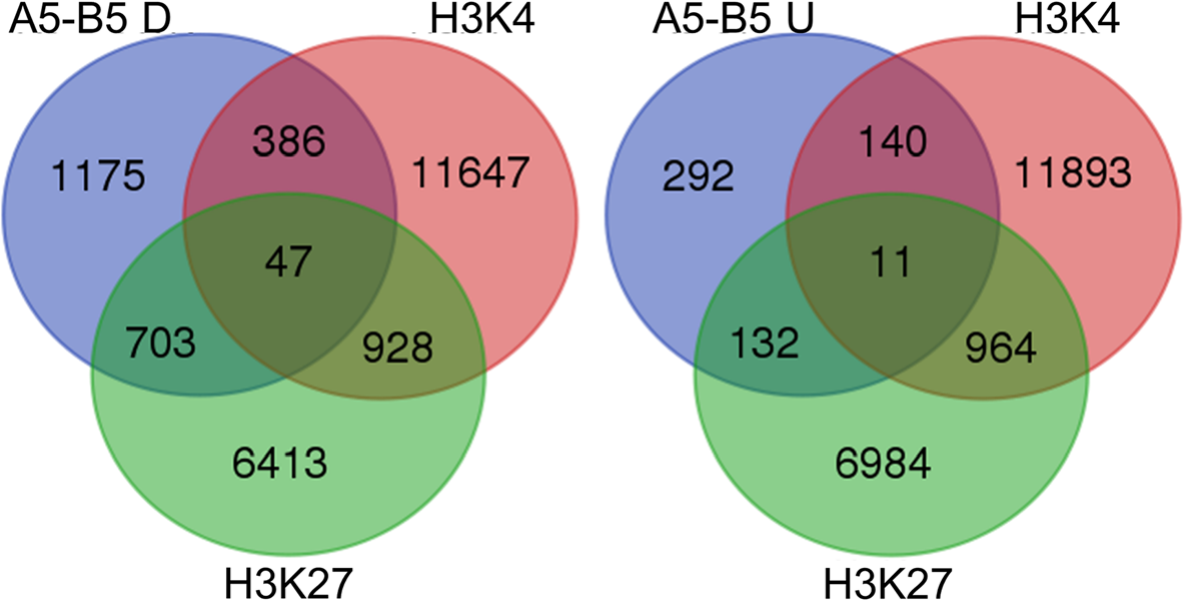
Venn diagram detailing DEGs enriched for H3K4me^3^ or H3K27me^3^ at mature pollen stage

To get more information of gene expression regulation in pollen development, we characterized the epigenetic control during pollen development by performing ChIP-seq on the fertile floral buds at mature pollen stage. The specific antibodies against trimethylated H3K4, a typical histone modification pattern characterizing active chromatin, and trimethylated H3K27 which is proposed to inhibit transcription, were used. A total of 13,008 and 8091 genes were found enriched for H3K4me^3^ and H3K27me^3^, respectively. Combined with RNA-seq transcriptome analysis, we found that 433 genes enriched for H3K4me^3^ and 750 genes enriched for H3K27me^3^ were down-regulated in the sterile floral buds, while 151 genes enriched for H3K4me^3^ and 143 genes enriched for H3K27me^3^ were up-regulated in the sterile floral buds at mature pollen stage. And there were 47 down-regulated and 11 up-regulated genes covalently modified by both H3K4me^3^ and H3K27me^3^ (Fig. 10). GO classifications were obtained to investigate the functions of these genes (Additional file 2: Table S7). It was worth mentioning that enriched GO terms in the group of down-regulated genes marked with H3K27me^3^ were associated with pollen tube development, cell tip growth, cell wall modification, pollination and reproductive developmental process suggesting an important role of H3K27me^3^ during pollen tube growth (Additional file 6: Figure S6). The molecular functions of these targets concentrated mainly on ATP binding, metal ion binding, for example, calcium ion binding and hydrolase activity, especially pectinesterase activity and polygalacturonase activity (Additional file 7: Figure S7).

Besides, 41 and 25 pollen development-related genes covalently modified by H3K4me^3^ and H3K27me^3^ were selected respectively, according to the published reports of Arabidopsis mutants affecting anther or pollen development as well as pollen germination or pollen tube growth (Table 4). The majority of these genes were involved in pollen maturation, pollen germination and pollen tube growth, over-represented by the genes encoding calmodulin-binding proteins. Those late-expressed genes enriched for H3K4 me^3^ participated in biological processes including glucose catabolic process, protein phosphorylation, nucleotide-sugar transport, oxylipin biosynthetic process and so on. While for late-expressed genes enriched for H3K27me^3^, they were mainly involved in auxin signaling transduction, calcium ion transport, membrane trafficking and regulation of transcription. Several early-expressed genes during anther and pollen development were also found to be marked with H3K4me^3^ or H3K27me^3^, over-represented by genes required for pollen exine formation. It is noticed that H3K27me^3^ targeted three key tapetum development-related genes including two transcription factor-encoding genes, *BrMS1*, *BrAMS* and Bra000615, the ortholog of Arabidopsis *CEP1*. Among these 66 genes, Bra014776 was the only example of a bivalent gene displayed enrichment for both H3K4me^3^ and H3K27me^3^.

**Table 4:** Pollen development-related genes covalently modified by H3K4me^3^ and H3K27me^3^

Classification	*Brassica rapa*			Type	Arabidopsis			
	Locus	Fold enrichment	FDR (%)		Locus	Gene name	Description	References
Meiosis process	Bra015676	6.35	2.19	H3K4	AT1G77320	*MEI1*	BRCT-domain-containing protein	[38]
	Bra029127	7.58	2.16	H3K4	AT5G52290	*SHOC1*	protein with similarity to XPF endonucleases	[39]
	Bra017718	6.02	4.73	H3K4	AT4G35520	*MLH3*	DNA mismatch repair protein	[40]
	Bra000955	9.92	1.61	H3K4	AT4G01370	*MAPK4*	MAP kinase	[41]
	Bra019444	2.27	11.76	H3K27	AT3G43210	*TES*	ATP binding microtubule motor family protein	[42]
	Bra023796	9.7	44.54	H3K27	AT3G22880	*DMC1*	DNA repair (Rad51) family protein	[43]
	Bra001890	7.24	23.26	H3K27	AT3G22880			
Tapetum development	**Bra009277**	10.25	1.91	H3K4	AT5G07280	*EMS1*	leucine-rich repeat receptor protein kinase	[44]
	**Bra002401**	5.8	11.36	H3K27	AT5G22260	*MS1*	PHD-finger motif transcription factor	[17]
	**Bra000615**	4.68	12.43	H3K27	AT5G50260	*CEP1*	papain-like cysteine protease	[45]
	**Bra013041**	6.24	11.48	H3K27	AT2G16910	*AMS*	basic helix-loop helix transcription factor	[16]
Pollen exine formation	**Bra014776**	10.36	1.52	H3K4	AT3G55090	*ABCG16*	ABCG half-transporter	[22]
	**Bra024796**	9.47	1.76	H3K4	AT2G02970	*APY6*	putative apyrase	[46]
	Bra017147	7.5	1.52	H3K4	AT1G02050	*LAP6*	chalcone and stilbene synthase family	[25]
	Bra008832	15.49	12.07	H3K4	AT5G13390	*NEF1*	membrane protein	[47]
	Bra023407	15.38	5.03	H3K4	AT5G13390			
	Bra034054	6.58	2.42	H3K4	AT3G09090	*DEX1*	membrane associated protein	[48]
	Bra029746	6.58	2.09	H3K4	AT3G09090			
	Bra038909	6.69	2.54	H3K4	AT3G51610	*NPU*	membrane protein	[49]
	**Bra014776**	8.58	11.76	H3K27	AT3G55090	*ABCG16*	ABCG half-transporter	[22]
	**Bra025595**	7.8	11.49	H3K27	AT5G40260	*RPG1*	a member of the MtN3/saliva gene family	[50]
	**Bra013442**	4.12	15.6	H3K27	AT4G20050	*QRT3*	polygalacturonase	[51]
Pollen maturation	Bra029700	9.92	1.51	H3K4	AT3G08590	*IPGAM2*	phosphoglycerate mutase	[52]
	Bra022067	7.76	1.59	H3K4	AT3G01780	*TPLATE*	cytokinesis protein	[53]
	Bra037331	6.58	1.55	H3K4	AT4G00330	*CRCK2*	calmodulin-binding receptor-like cytoplasmtic kinase	[54]
	Bra001510	5.46	2.32	H3K4	AT3G13530	*MAP3KE1*	epsilon protein kinase	[55]
	Bra001358	4.9	3.06	H3K4	AT3G10405	*–*	vacuolar acid trehalase	[54]
	**Bra026579**	4.57	5.65	H3K4	AT2G02810	*UTR1*	multitransmembrane hydrophobic protein	[56]
	Bra008037	5.02	2.43	H3K4	AT1G72520	*LOX4*	PLAT/LH2 domain-containing lipoxygenase family protein	[57]
	Bra024316	5.02	3.98	H3K4	AT5G64630	*FAS2*	chromatin assembly factor-1 p60 subunit	[58]
	Bra029541	8.88	1.64	H3K4	AT4G04970	*GSL1*	callose synthase	[59]
	**Bra008722**	3.01	17.63	H3K27	AT5G15100	*PIN8*	auxin transporter	[60]
	Bra008615	9.47	14.76	H3K27	AT5G16530	*PIN5*	an atypical member of the PIN family	[61]
	**Bra039940**	7.91	11.59	H3K27	AT2G22950	*ACA7*	putative auto-regulated Ca^2+^-ATPase	[62]
	**Bra033961**	3.57	15.97	H3K27	AT1G68090	*ANN5*	calcium-binding protein	[63]
	**Bra007536**	6.69	11.27	H3K27	AT3G60460	*DUO1*	R2R3 MYB transcription factor	[64]
	Bra003413	5.57	14.1	H3K27	AT3G60460			
Pollen maturation and anther dehiscence	Bra002816	11.48	1.82	H3K4	AT5G56450	*PM-ANT*	mitochondrial substrate carrier family protein	[65]
	Bra004869	6.8	11.27	H3K27	AT2G44810	*DAD1*	alpha/beta hydrolases superfamily protein	[66]
Pollen development and tube growth	Bra040162	5.35	2.09	H3K4	AT4G17530	*RAB1C*	Rab GTPase	[67]
	Bra009268	5.35	2.74	H3K27	AT5G07370	*IPK2A*	inositol polyphosphate kinase	[68]
	**Bra015643**	3.68	14.95	H3K27	AT1G77980	*AGL66*	MADS transcription factor	[69]
Pollen germination and tube growth	Bra004738	5.24	4.14	H3K4	AT2G43040	*NPG1*	calmodulin-binding protein	[70]
	Bra010936	4.9	5.3	H3K4	AT1G27460	*NPGR1*	calmodulin-binding protein	[70]
	Bra011041	4.79	3.81	H3K4	AT4G28600	*NPGR2*	calmodulin-binding protein	[70]
	Bra010358	4.57	5.02	H3K4	AT4G28600			
	Bra032785	4.57	5.35	H3K4	AT1G24620	*CML25*	EF-hand calcium-binding protein	[71]
	Bra011622	10.36	1.5	H3K4	AT4G35540	*PTF2*	TFIIB-related protein	[72]
	Bra039141	6.46	1.52	H3K4	AT3G01150	*PTB1*	polypyrimidine tract-binding protein	[73]
	Bra003466	6.35	2.47	H3K4	AT3G61710	*ATG6*	autophagy protein	[74]
	Bra005376	7.91	1.49	H3K4	AT2G34980	*SETH1*	phosphatidylinositol-glycan synthase subunit	[75]
	Bra031332	4.57	5.25	H3K4	AT3G22200	*POP2*	gamma-aminobutyrate transaminase	[76]
	Bra009450	10.59	1.52	H3K4	AT5G04480	*BUP*	putative glycosyltransferase	[77]
	Bra000950	9.81	1.43	H3K4	AT4G01220	*MGP4*	Rhamnogalacturonan II xylosyltransferase	[78]
	Bra015010	8.92	1.51	H3K4	AT4G38430	*ROPGEF1*	guanine nucleotide exchange factor	[79]
	Bra023489	6.24	2.66	H3K4	AT5G14850	*APTG1*	mannosyltransferase	[80]
	**Bra008404**	5.91	1.53	H3K4	AT1G79250	*AGC1.7*	AGC kinase	[81]
	Bra014529	5.46	2.83	H3K4	AT3G59760	*ATCS-C*	O-acetylserine lyase	[82]
	**Bra008733**	10.81	11.6	H3K27	AT5G14870	*CNGC18*	a member of the cyclic nucleotide gated channel family	[83]
	Bra007785	7.91	14.93	H3K27	AT2G25600	*SPIK*	member of the Shaker family potassium ion (K^+^) channel	[84]
	**Bra000105**	8.69	11.79	H3K27	AT2G38910	*CPK20*	calcium dependent protein kinase	[85]
	**Bra015442**	6.91	11.81	H3K27	AT1G05580	*CHX23*	member of putative Na^+^/H^+^ antiporter family	[86]
	**Bra025052**	4.79	14.1	H3K27	AT5G45810	*CIPK19*	CBL-interacting protein kinase	[87]
	**Bra022172**	4.35	15.47	H3K27	AT3G16640	*TCTP1*	a protein homologous to translationally controlled tumor protein	[88]
	Bra027968	4.01	17.49	H3K27	AT1G54560	*XIE*	myosin family protein with DIL domain-containing protein	[89]
	**Bra008656**	4.01	14.78	H3K27	AT5G16020	*GEX3*	plasma membrane localized protein	[90]

Genes were selected on the basis of previous reports of *Arabidopsis* mutants affecting anther or pollen development as well as pollen germination or pollen tube growth. The Brassica ID in bold means the gene showed differential expressional levels between the fertile and sterile floral buds at mature pollen development in our RNA-seq analysis

## Discussion

In our previous analysis, we have concluded that the defect in male meiotic cytokinesis leads to male sterility in ‘Bcajh97-01A’ [34]. As it is well known, a characteristic feature of male meiotic cytokinesis is the abundant presence of callose. In this study, cytochemical staining for callose during the meiosis process further supported our result. Among 12 genes encoding putative callose synthase identified in Arabidopsis, *GSL1*, *GSL5* and *GSL2* have been shown to be involved in callose synthesis during microsporogenesis. Mutants of these genes display defective callose synthesis and exine patterning [59, 91]. On the other hand, callase mixture secreted by the tapetum is responsible for callose degradation, represented by the *A6* gene encoding β-1, 3-glucanase. Knockout or knockdown of β-1, 3-glucanase genes in Arabidopsis, *B. napus* and rice frequently caused defective callose degradation and ultimately led to male sterility [92, 93]. In addition, *AtMYB80* and *CDM1* have been reported to play an important role in regulation of callose metabolism during microsporogenesis [13, 94]. Mutation in *CDM1* disturbs the normal callose metabolism by down-regulating of *AtGSL5* and *AtGSL2* and activating in advance of *A6*. At last, the *cdm1* mutant is completely male sterile resulting from delayed meiotic cytokinesis and microspore degeneration. In our analysis, although ortholog of Arabidopsis *CDM1* was dramatically down-regulated and the phenotype of pollen abortion was similar to that of *cdm1*, the regulation of callose metabolic pathways may be different as no one callose synthase gene was affected in the sterile line at early pollen development. In addition, all the expression of early-expressed β-1, 3-glucanase genes seemed to be down-regulated first and then remarkably up-regulated. However, the expression change of β-1, 3-glucanase genes was different in *cdm1*. Although Arabidopsis has 55 genes encoding putative β-1, 3-glucanases, only *A6* encoding protein was thought to be part of the callose enzyme complex [95]. However, a recent research has speculated that three other β-1, 3-glucanase genes (At3g24330, At3g55780, and At3g61810) might participate in callose dissolution during microsporogenesis and pollen development [94]. In this study, we identified two more groups of β-1, 3-glucanase genes showing changed expression in the sterile floral buds. All seven early-expressed genes displayed similar expressional changes in the sterile floral buds. And their Arabidopsis orthologs (At4g14080, At3g61810 and At3g23770) were closely related with each other according to phylogenetic analysis and belong to the expression group K which includes β-1, 3-glucanase genes highly specific to anther [95]. All these suggested they might play a similar role in degradation of callose wall surrounding the tetrads.

Another particular phenotype of ‘Bcajh97-01A’, compared with *cdm1*, was the disorder development of tapetum. In *cdm1*, the tapetum layer appeared normal and genes known to be important for tapetum development were not dramatically affected, while the tapetum layer of ‘Bcajh97-01A’ went on premature PCD followed by abnormal development of tapetosomes and elaioplasts. And a series of regulatory genes for tapetum development were altered, including *BrDYT1*, *BrAMS*, *BrMYB80* and *BrMS1*. In Brassicaceae species, the main pollen exine materials are stored and transferred by tapetosomes, the lipid accumulating ER-derived spherical organelles in tapetal cells and elaioplasts, the specialized plastid derived from proplastids [96–98]. In Arabidopsis mutants, *ams* and *ms1*, as well as a *B. napus* male sterile mutant, abnormal tapetal development caused decrease or even absence of tapetosomes and elaioplasts leading to disordered pollen wall formation [17, 99, 100]. Studies have shown that either premature or retarded tapetal PCD could induce defects in typical tapetosomes and elaioplasts formation [45, 101]. Here, combined with our phenotype observation, we presumed that abnormal development of tapetosomes and elaioplasts caused by premature tapetal PCD led to defective pollen exine formation in the sterile ‘Bcajh97-01A’ line.

Unlike exine development which is mainly controlled by tapetum cell, intine formation has long been assumed to be mainly contributed by microspores. Although the exact mechanism is not clear, pectin metabolism-associated genes have been reported to be essential for pollen wall development [8]. As with other transcriptomic analyses related with male sterility, a high proportion of cell wall modification-related genes involved in polysaccharide metabolism, including *PME*s, *PMEI*s, *PG*s and *PLL*s, were significantly down-regulated in the sterile line in our study [102–104]. In Arabidopsis, 14 of 66 putative *PME*s are specifically expressed in pollen grains or pollen tubes [105, 106]. Two of these *PME*s, *AtVDG1* and *AtPPME1* have been reported to be essential for pollen tube elongation and stability [29, 107]. And, *AtPME48*, the second most expressed *PME* in dry and imbibed pollen grains, was found to be closely associated with pollen grain germination by influencing remodeling the homogalacturonan of the intine cell wall [30]. Here in our study, a total of 13 *PME*s that showed particularly high expression at mature pollen stage, including orthologs of *AtVDG1* and *AtPPME1*, were down-regulated in the sterile floral buds. These genes are presumed to be involved in pollen maturation, pollen germination and pollen tube growth affecting development of pollen intine. Recently, one of these *PME*s has been proved to function in intine construction by our lab [108]. As for those 22 PMEIs highly expressed in pollen, they are presumed to be key regulators of cell wall stability during pollen tube growth by interacting with PMEs, although more details about the molecular mechanism of interaction needs to be clarified [109–112]. In *B. rapa*, several PG genes have been demonstrated to regulate different aspects of pollen development in our previous studies, with *BcMF2* affecting pollen intine, *BcMF6* affecting microsporogenesis and pollen maturation, and *BcMF9* affecting both intine and exine formation [113–115]. In fact, two down-regulated *PG*s found in our study, that were Bra032138 and Bra037005, have been confirmed to be pollen-expressed and the latter one was demonstrated to participate in pollen wall construction by modulating intine information [31, 116]. The overlapping but distinct expression patterns for most of these cell wall hydrolytic enzyme-coding genes implied complicated roles they may play during the plant reproductive processes.

Transcriptional regulation plays pivotal roles in the control of gene expression in plants. In Arabidopsis, 612 of 1350 predicted TFs were expressed in developing male gametophytes and a number of TFs have been identified as key regulators during plant reproductive development as Khurana et al. summarized [117]. Here in our study, 493 predicted TFs showed changed expression level in the sterile line compared with the fertile line representing candidates for transcriptional regulators of male gametophyte development. The analysis of stage-specific expression profiling of these TFs during floral bud development helps to make a prediction of their gene functions. One of these TFs, Bra016531, a novel MYB gene was indicated to be related with tapetum development and function in our study.

**Fig. 11:**
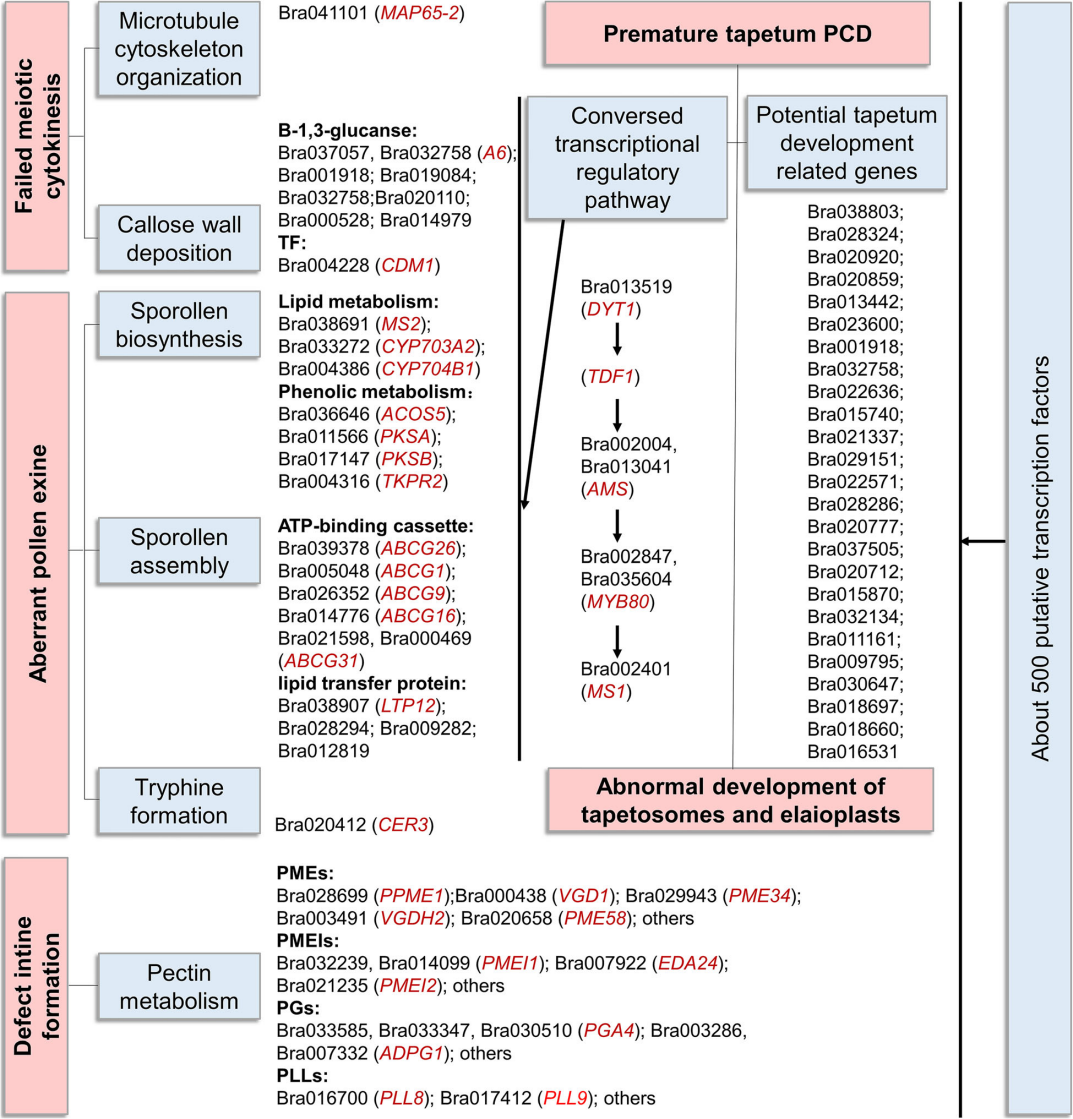
A summary of genes involved in various processes of pollen wall development in *Brassica rapa*

Comparative transcriptome analysis of the sterile and fertile floral buds of ‘Bcajh97-01A/B’ at five typical pollen developmental stages identified a great deal of differentially expressed genes. On the basis of advances in Arabidopsis, and combined with detailed phynotype observation, we make a summary of genes involved in various processes of pollen wall development in *B. rapa* (Fig. 11).

Besides proteins functioning as transcriptional regulators, epigenetic modifications of DNA and associated proteins have been identified as triggers for gene activity. Histone lysine methylation has emerged as a critical player in the regulation of gene expression by controlling the chromatin state in many organisms [118, 119]. The deposition of H3K4me^3^ have been reported to be closely related with plant reproductive development [120, 121]. But how H3K4me^3^ and H3K27me^3^ deposition participate in pollen development still remains largely unknown. In this study, a large number of H3K4me^3^ or H3K27me^3^ enriched DEGs and pollen development-related genes were identified providing materials for further analysis of epigenetic control on gene expression during pollen development. We have also noticed that both H3K4me^3^ and H3K27me^3^ targeted several key genes involved in tapetum development and pollen exine formation suggesting vital roles of H3K4me^3^ and H3K27me^3^ deposition in regulation of pollen wall construction.

## Conclusions

In this study, transcriptome profiling on the floral buds of a GMS line with aberrant meiotic cytokinesis allowed the generating of stage-specific gene expression profiles and identification of candidate genes underlying pollen wall formation and genic male sterility. With the application of ChIP-sequencing on floral buds, a new sight into epigenetic control on gene expression during pollen development and pollen wall formation was provided. The data presented here provides a powerful platform for future functional and molecular regulation research of pollen wall formation and pollen development in *B. rapa*.

## Methods

### Plant materials and growth conditions

‘Bcajh97-01A/B’, a Chinese cabbage (*B. rapa* ssp. *chinensis* cv. Aijiaohuang) genic male sterile A/B line, was bred by Laboratory of Cell and Molecular Biology, Institute of Vegetable Science, Zhejiang University and was cultivated in the experimental farm of Zhejiang University, Hangzhou, China. Floral buds at different pollen developmental stages (Stage I, pollen mother cell stage; Stage II, tetrad stage; Stage III, uninucleate microspore stage; Stage IV, binucleate microspore stage; and Stage V, mature pollen stage) were named as A1 to A5 in ‘Bcajh97-01A’ plants and B1 to B5 in ‘Bcajh97-01B’ plants according to the division previously described by Huang et al. [122].

All Arabidopsis (ecotype Colombia) plants were grown in a 22 ± 1 °C growth chamber under long-day conditions (16 h light/8 h dark).

### Morphological and cytological observation

Pollen grains were observed by a scanning electron microscope as previously described [123]. Aniline blue staining was performed as described [13] and DAPI staining was made as previously described [31]. Both the micrographs of callose fluorescence and chromatin fluorescence were captured with a Leica DMLB fluorescence microscope under UV light. For transmission electron microscopy (TEM) analysis, the procedures were as described in reference [123] and the samples for TEM observation were also used for semi-thin section observation.

### RNA sequencing, transcriptome assembly and annotation of Unigenes

Total RNA was isolated from both the sterile and fertile floral buds at five pollen developmental stages according to the instructions of the TRIzol kit (Invitrogen, USA) and sent for RNA-seq. After enrichment using NEBNext Poly (A) mRNA Magnetic Isolation Module (NEB, E7490), mRNA was used to construct cDNA libraries using NEBNext mRNA Library Prep Master Mix Set for Illumina (NEB, E6110) and NEBNext Multiplex Oligos for Illumina (NEB, E7500). The high-quality libraries were then sequenced by Biomarker Technologies (http://www.biomarker.com.cn) on the Illumina HiSeqTM2500. After filtering out low quality data, over 14,500,000 clean reads were assembled by running Trinity, resulting 25,509,284 contigs. Then all contigs were clustering to transcripts and Unigenes according to the pair-end information and similarities between contigs.

### GO and pathway analysis

GO annotations of the Unigenes and DEGs were determined using agriGO [124]. Then, the results were submitted to WEGO to obtain the GO classification graph [125]. GO analysis applied a hypergeometric test to identify significantly enriched GO terms in DEGs in comparison to the transcriptome generated by this study. We chose the Bonferroni to do the multi-test correction, set 5 as minimum number of mapping entries, and we used a corrected-*p* value ≤0.05 as the threshold value. GO term (*P* ≤ 0.05) was defined as significantly enriched GO term. For pathway analysis, the DEGs were submitted to KEGG Automatics Annotation Server (KAAS) and classified with the single directional best hit (SBH) method [126]. Then, the results were submitted to KEGG Mapper to obtain the KEGG map.

### qRT-PCR analysis

Total RNA was extracted from five different tissues including roots, stems, leaves, inflorescences and siliques using TRIzol Reagent (nvitrogen, USA) and reverse transcribed into the first strand of cDNA using PrimerScript RT reagent Kit (Takara, Dalian, China). For qRT-PCR analysis of Bra016531, *BcUBC10* was used as the reference gene and qRT-PCR reaction was performed using the SYBR® Premix Ex Taq™ Kit (TaKaRa, Dalian, China) in a CFX96 Real-Time System (Bio-Rad, California, USA) with the gene-specific pairs (Additional file 2: Table S8). For each sample, three biological replicates were conducted with three technical replicates, and the results were calculated using the 2^-ΔΔCt^ method [127].

### Promoter analysis

A 996-bp genomic DNA fragment containing nucleotide sequence from 976-bp upstream of the Bra016531 start codon to the first 20 bp of the first exon was amplified (Additional file 8: Figure S5) and subcloned upstream of the *GUS* reporter gene in the pBI101 vector (Dalian, China) and was introduced into wild-type Arabidopsis using the *Agrobacterium*-mediated floral-dip method [128]. For observation of Bra016531pro::*GUS* expression, T_1_ and T_2_ plants from more than 10 independent transgenic lines were stained with GUS staining solution as described by Kim et al. [129]. Moreover, the detailed examine in anther cells was performed as described by Lyu et al. [31].

### Library construction and sequencing for ChIP

Floral buds of the fertile line at mature pollen stage were sent to Cloud-seq Technologies (http://www.cloud-seq.com.cn) for ChIP-seq. Chromatin Immunoprecipitation was performed according to Wamstad et al. [130]. The yield of ChIPed DNA was determined via Quant IT fluorescence assay (Life Technologies) and enrichment efficiencies of ChIP reactions were evaluated by qPCR. Illumina sequencing libraries were generated with NEBNext® Ultra™ DNA Library Prep Kit (New England Biolabs) by following the manufacturer’s manual. The library quality was determined by using Agilent 2100 Bioanalyzer (Agilent), and then, subjected to high-throughput 150 base paired-end sequencing on Illumina Hiseq sequencer according to the manufacturer’s recommended protocol.

After filtering out low quality data, there are 8,850,598 reads from the control sample “Input” mapped to *B. rapa* genome (version 1.5). As for sample “H3K4me^3^” and “H3K27me^3^”, the numbers were 10,222,458 and 10,177,636 respectively. For enriched-region (peak) identification (peak calling), the Model-based Analysis of ChIP-seq (MACS) algorithm was used [131]. And then, bedtools software was used for peak annotation [132].

## Additional files

Additional file 1: **Figure S1.** The length distribution of assembled *Brassica rapa* contigs. (TIF 1833 kb)

Additional file 2: **Table S1.** Summary of *Brassica rapa* assembled transcriptome. **Table S2.** Statistics of annotation results for *Brassica rapa* Unigenes. **Table S3.** The expression of DEGs in floral buds of the fertile and sterile lines (RPKM value). **Table S4.** The significantly enriched Gene Ontology (GO) terms by DEGs at each stage. **Table S5.** The Kyoto Encyclopedia of Genes and Genomes (KEGG) pathway by DEGs at each stage. **Table S6.** Expressional changes of putative transcription factors. **Table S7.** GO analysis of DEGs enriched for H3K4me^3^ or H3K27me^3^. **Table S8.** Primers used in this article. (XLSX 971 kb)

Additional file 3: **Figure S2.** The length distribution of assembled *Brassica rapa* Unigenes. (TIF 2354 kb)

Additional file 4: **Figure S3.** COG functional classification of all Unigene sequences. (TIF 1450 kb)

Additional file 5: **Figure S4.** GO classification of *Brassica rapa* Unigenes. (TIF 5347 kb)

Additional file 6: **Figure S5.** Information of ProBra016531. (TIF 464 kb)

Additional file 7: **Figure S6.** Significantly enriched Biological Processes GO terms in the group of down-regulated genes marked with H3K27me^3^. (TIF 2813 kb)

Additional file 8: **Figure S7.** Significantly enriched Molecular Functions GO terms in the group of down-regulated genes marked with H3K27me^3^. (TIF 4779 kb)

## Abbreviations

BP: Biological processes
CC: Cellular components
ChIP-seq: ChIP sequencing
COG: Clusters of Orthologous Groups
DAPI: 4′, 6-diamidino-2-phenylindole
DEGs: Differentially expressed genes
FDR: False discovery rate
GMS: Genic male sterility
GO: Gene ontology
H3K27 me^3^: trimethylated histone H3 lysine 27
H3K4 me^3^: trimethylated histone H3 lysine 4
KAAS: KEGG automatics annotation server
KEGG: Kyoto encyclopedia of genes and genome
MACS: Model-based Analysis of ChIP-seq
MF: Molecular functions
NR: Non-redundant protein sequences
PCD: Programmed cell death
PGs: Polygalacturonases
PLLs: Pectate lyases like proteins
PMEI: Pectin methylesterase inhibitor protein
PMEs: Pectin methylesterases
RNA-seq: RNA sequencing
SBH: Single directional best hit
SEM: Scanning electron microscopy
TEM: Transmission electron microscopy
TFs: Transcription factors

## References

[1] Gómez JF, Talle B, Wilson ZA (2015). Anther and pollen development: A conserved developmental pathway. J Inter Plant Biol..

[2] Jiang J, Zhang Z, Cao J (2013). Pollen wall development: the associated enzymes and metabolic pathways. Plant Biol..

[3] Scott RJ, Spielman M, Dickinson HG (2004). Stamen Structure and Function. Plant Cell..

[4] Ariizumi T, Toriyama K (2012). Genetic Regulation of Sporopollenin Synthesis and Pollen Exine. Annu Rev Plant Biol..

[5] Wilson ZA, Zhang DB (2009). From Arabidopsis to rice: pathways in pollen development. J Exp Bot..

[6] Schnurr JA, Storey KK, Jung HJG, Somers DA, Gronwald JW (2006). UDP-sugar pyrophosphorylase is essential for pollen development in Arabidopsis. Planta..

[7] Blackmore S, Wortley AH, Skvarla JJ, Rowley JR (2007). Pollen wall development in flowering plants. New Phytol..

[8] Shi J, Cui M, Yang L, Kim YJ, Zhang D (2015). Genetic and Biochemical Mechanisms of Pollen Wall Development. Trends Plant Sci..

[9] Hafidh S, Fíla J, Honys D (2016). Male gametophyte development and function in angiosperms: a general concept. Plant Reprod..

[10] Zhang D, Shi J, Yang X (2016). Role of Lipid Metabolism in Plant Pollen Exine Development. Subcell Biochem..

[11] Xu T, Zhang C, Zhou Q, Yang ZN (2016). Pollen wall pattern in Arabidopsis. Sci Bull..

[12] Zhang W, Sun Y, Timofejeva L, Chen C, Grossniklaus U, Ma H (2006). Regulation of Arabidopsis tapetum development and function by *DYSFUNCTIONAL TAPETUM1* (*DYT1*) encoding a putative bHLH transcription factor. Development..

[13] Zhang ZB, Zhu J, Gao JF, Wang C, Li H, Li H, Zhang HQ, Zhang S, Wang DM, Wang QX, Huang H, Xia HJ, Yang ZN (2007). Transcription factor *AtMYB103* is required for anther development by regulating tapetum development, callose dissolution and exine formation in Arabidopsis. Plant J..

[14] Gu JN, Zhu J, Yu Y, Teng XD, Lou Y, Xu XF, Liu J, Yang ZN (2014). *DYT1* directly regulates the expression of *TDF1* for tapetum development and pollen wall formation in Arabidopsis. Plant J..

[15] Zhu J, Chen H, Li H, Gao JF, Jiang H, Wang C, Guan YF, Yang ZN (2008). *Defective in Tapetal development and function 1* is essential for anther development and tapetal function for microspore maturation in Arabidopsis. Plant J..

[16] Xu J, Yang C, Yuan Z, Zhang D, Gondwe MY, Ding Z, Liang W, Zhang D, Wilson ZA (2010). The ABORTED MICROSPORES regulatory network is required for postmeiotic male reproductive development in *Arabidopsis thaliana*. Plant Cell..

[17] Yang C, Vizcay-Barrena G, Conner K, Wilson ZA (2007). *MALE STERILITY 1* is required for tapetal development and pollen wall biosynthesis. Plant Cell..

[18] Huang MD, Chen TL, Huang AH (2013). Abundant type III lipid transfer proteins in Arabidopsis tapetum are secreted to the locule and become a constituent of the pollen exine. Plant Physiol..

[19] Choi H, Jin JY, Choi S, Hwang JU, Kim YY, Suh MC, Lee Y (2011). An ABCG/WBC-type ABC transporter is essential for transport of sporopollenin precursors for exine formation in developing pollen. Plant J..

[20] Choi H, Ohyama K, Kim YY, Jin JY, Lee SB, Yamaoka Y, Muranaka T, Suh MC, Fujioka S, Lee Y (2014). The role of Arabidopsis ABCG9 and ABCG31 ATP binding cassette transporters in pollen fitness and the deposition of steryl glycosides on the pollen coat. Plant Cell..

[21] Quilichini TD, Samuels AL, Douglas CJ (2014). ABCG26-mediated polyketide trafficking and hydroxycinnamoyl spermidines contribute to pollen wall exine formation in Arabidopsis. Plant Cell..

[22] Yadav V, Molina I, Ranathunge K, Castillo IQ, Rothstein SJ, Reed JW (2014). ABCG transporters are required for suberin and pollen wall extracellular barriers in Arabidopsis. Plant Cell..

[23] de Azevedo Souza C, Kim SS, Koch S, Kienow L, Schneider K, McKim SM, Haughn GW, Kombrink E, Douglas CJ (2009). A novel fatty Acyl-CoA Synthetase is required for pollen development and sporopollenin biosynthesis in Arabidopsis. Plant Cell..

[24] Morant M, Jorgensen K, Schaller H, Pinot F, Moller BL, Werck-Reichhart D, Bak S (2007). CYP703 is an ancient cytochrome P450 in land plants catalyzing in-chain hydroxylation of lauric acid to provide building blocks for sporopollenin synthesis in pollen. Plant Cell..

[25] Dobritsa AA, Lei Z, Nishikawa S, Urbanczykwochniak E, Huhman DV, Preuss D, Sumner LW (2010). *LAP5* and *LAP6* encode anther-specific proteins with similarity to chalcone synthase essential for pollen exine development in Arabidopsis. Plant Physiol..

[26] Kim SS, Grienenberger E, Lallemand B, Colpitts CC, Kim SY, Souza Cde A, Geoffroy P, Heintz D, Krahn D, Kaiser M, Kombrink E, Heitz T, Suh DY, Legrand M, Douglas CJ (2010). *LAP6*/*POLYKETIDE SYNTHASE A* and *LAP5*/*POLYKETIDE SYNTHASE* B encode hydroxyalkyl alpha-pyrone synthases required for pollen development and sporopollenin biosynthesis in *Arabidopsis thaliana*. Plant Cell..

[27] Chen W, Yu XH, Zhang K, Shi J, De Oliveira S, Schreiber L, Shanklin J, Zhang D (2011). *Male Sterile 2* encodes a plastid-localized fatty acyl carrier protein reductase required for pollen exine development in Arabidopsis. Plant Physiol..

[28] Grienenberger E, Kim SS, Lallemand B, Geoffroy P, Heintz D, Souza Cde A, Heitz T, Douglas CJ, Legrand M (2010). Analysis of *TETRAKETIDE α-PYRONE REDUCTASE* function in *Arabidopsis thaliana* reveals a previously unknown, but conserved, biochemical pathway in sporopollenin monomer biosynthesis. Plant Cell..

[29] Jiang L, Yang SL, Xie LF, Puah CS, Zhang XQ, Yang WC, Sundaresan V, Ye D (2005). *VANGUARD1* encodes a pectin methylesterase that enhances pollen tube growth in the Arabidopsis style and transmitting tract. Plant Cell..

[30] Leroux C, Bouton S, Kiefer-Meyer MC, Fabrice TN, Mareck A, Guenin S, Fournet F, Ringli C, Pelloux J, Driouich A, Lerouge P, Lehner A, Mollet JC (2015). *PECTIN METHYLESTERASE48* is involved in Arabidopsis pollen grain germination. Plant Physiol..

[31] Lyu M, Yu Y, Jiang J, Song L, Liang Y, Ma Z, Xiong X, Cao J (2015). *BcMF26a* and *BcMF26b* Are Duplicated Polygalacturonase Genes with Divergent Expression Patterns and Functions in Pollen Development and Pollen Tube Formation in *Brassica campestris*. PloS one..

[32] Jiang J, Yao L, Yu Y, Liang Y, Jiang J, Ye N, Miao Y, Cao J (2014). *PECTATE LYASE-LIKE 9* from *Brassica campestris* is associated with intine formation. Plant Sci..

[33] Huang L, Cao J, Ye W, Liu T, Jiang L, Ye Y (2008). Transcriptional differences between the male-sterile mutant bcms and wild-type *Brassica campestris ssp. chinensis* reveal genes related to pollen development. Plant Biol..

[34] Huang L, Ye W, Liu T, Cao J (2009). Characterization of the male-sterile line Bcajh97-01A/B and identification of candidate genes for genic male sterility in Chinese cabbage-pak-choi. Oncol Rep..

[35] Cavell AC, Lydiate DJ, Parkin IA, Dean C, Trick M (1998). Collinearity between a 30-centimorgan segment of *Arabidopsis thaliana* chromosome 4 and duplicated regions within the *Brassica napus* genome. Genome..

[36] Wijeratne AJ, Zhang W, Sun Y, Liu W, Albert R, Zheng Z, Oppenheimer DG, Zhao D, Ma H (2007). Differential gene expression in Arabidopsis wild-type and mutant anthers: insights into anther cell differentiation and regulatory networks. Plant J..

[37] Alves-Ferreira M, Wellmer F, Banhara A, Kumar V, Riechmann JL, Meyerowitz EM (2007). Global expression profiling applied to the analysis of Arabidopsis stamen development. Plant Physiol..

[38] Mathilde G, Ghislaine G, Daniel V, Georges P (2003). The Arabidopsis *MEI1* gene encodes a protein with five BRCT domains that is involved in meiosis-specific DNA repair events independent of SPO11-induced DSBs. Plant J..

[39] Macaisne N, Novatchkova M, Peirera L, Vezon D, Jolivet S, Froger N, Chelysheva L, Grelon M, Mercier R (2008). SHOC1, an XPF endonuclease-related protein, is essential for the formation of class I meiotic crossovers. Curr Biol..

[40] Jackson N, Sanchez-Moran E, Buckling E, Armstrong SJ, Jones GH (2006). Reduced meiotic crossovers and delayed prophase I progression in *AtMLH3*-deficient Arabidopsis. EMBO J..

[41] Zeng Q, Chen JG, Ellis BE (2011). *AtMPK4* is required for male-specific meiotic cytokinesis in Arabidopsis. Plant J..

[42] Yang CY, Spielman MColes JP, Li Y, Ghelani S, Bourdon V, Brown RC (2010). *TETRASPORE* encodes a kinesin required for male meiotic cytokinesis in Arabidopsis. Plant J..

[43] Muyt AD, Pereira L, Vezon D, Chelysheva L, Gendrot G, Chambon A, Lainéchoinard S, Pelletier G, Mercier R, Nogué F (2009). A High Throughput Genetic Screen Identifies New Early Meiotic Recombination Functions in *Arabidopsis thaliana*. PLoS Genet..

[44] Zhao DZ, Wang GF, Speal B, Ma H (2002). The *excess microsporocytes1* gene encodes a putative leucine-rich repeat receptor protein kinase that controls somatic and reproductive cell fates in the Arabidopsis anther. Genes Dev..

[45] Zhang D, Liu D, Lv X, Wang Y, Xun Z, Liu Z, Li F, Lu H (2014). The cysteine protease CEP1, a key executor involved in tapetal programmed cell death, regulates pollen development in Arabidopsis. Plant Cell..

[46] Yang J, Wu J, Romanovicz D, Clark G, Roux SJ (2013). Co-regulation of exine wall patterning, pollen fertility and anther dehiscence by Arabidopsis apyrases 6 and 7. Plant Physiol Bioch..

[47] Ariizumi T, Hatakeyama K, Hinata K, Inatsugi R, Nishida I, Sato S, Kato T, Tabata S, Toriyama K (2010). Disruption of the novel plant protein NEF1 affects lipid accumulation in the plastids of the tapetum and exine formation of pollen, resulting in male sterility in *Arabidopsis thaliana*. Plant J..

[48] Paxson-sowders DM, Dodrill CH, Owen HA, Makaroff CA (2001). DEX1, a Novel Plant Protein, Is Required for Exine Pattern Formation during Pollen Development in Arabidopsis. Plant Physiol..

[49] Chang HS, Yang ZN (2012). *NO PRIMEXIN AND PLASMA MEMBRANE UNDULATION* is essential for primexine deposition and plasma membrane undulation during microsporogenesis in Arabidopsis. Plant Physiol..

[50] Sun MX, Huang XY, Yang J, Guan YF, Yang ZN (2013). Arabidopsis *RPG1* is important for primexine deposition and functions redundantly with *RPG2* for plant fertility at the late reproductive stage. Plant Reprod..

[51] Rhee SY, Osborne E, Poindexter PD, Somerville CR (2003). Microspore separation in the *quartet 3* mutants of Arabidopsis is impaired by a defect in a developmentally regulated polygalacturonase required for pollen mother cell wall degradation. Plant Physiol..

[52] Zhao Z, Assmann SM (2011). The glycolytic enzyme, phosphoglycerate mutase, has critical roles in stomatal movement, vegetative growth, and pollen production in *Arabidopsis thaliana*. J Exp Bot..

[53] Van DD, Coutuer S, De RR, Bouget FY, Inzé D, Geelen D (2006). Somatic cytokinesis and pollen maturation in Arabidopsis depend on TPLATE, which has domains similar to coat proteins. Plant Cell..

[54] Boavida LC, Shuai B, Yu HJ, Pagnussat GC, Sundaresan V, Mccormick S (2009). A collection of Ds insertional mutants associated with defects in male gametophyte development and function in *Arabidopsis thaliana*. Genetics..

[55] Chaiwongsar S, Otegui MS, Jester PJ, Monson SS, Krysan PJ (2006). The protein kinase genes *MAP3K ɛ 1* and *MAP3K ɛ 2* are required for pollen viability in *Arabidopsis thaliana*. Plant J..

[56] Reyes F, León G, Donoso M, Brandizzí F, Weber APM, Orellana A (2010). The nucleotide sugar transporters AtUTr1 and AtUTr3 are required for the incorporation of UDP-glucose into the endoplasmic reticulum, are essential for pollen development and are needed for embryo sac progress in *Arabidopsis thaliana*. Plant J..

[57] Caldelari D, Wang G, Farmer EE, Dong X (2011). Arabidopsis *lox3lox4* double mutants are male sterile and defective in global proliferative arrest. Plant Mol Biol..

[58] Chen Z, Tan JL, Ingouff M, Sundaresan V, Berger F (2008). Chromatin assembly factor 1 regulates the cell cycle but not cell fate during male gametogenesis in *Arabidopsis thaliana*. Development..

[59] Enns LC, Kanaoka MM, Torii KU, Comai L, Okada K, Cleland RE (2005). Two callose synthases, GSL1 and GSL5, play an essential and redundant role in plant and pollen development and in fertility. Plant Mol Biol..

[60] Bosco CD, Dovzhenko A, Liu X, Woerner N, Rensch T, Eismann M, Eimer S, Hegermann J, Paponov IA, Ruperti B (2012). The endoplasmic reticulum localized PIN8 is a pollen-specific auxin carrier involved in intracellular auxin homeostasis. Plant J..

[61] Ding Z, Wang B, Moreno I, Dupláková N, Simon S, Carraro N, Reemmer J, Pěnčík A, Chen X, Tejos R (2012). ER-localized auxin transporter PIN8 regulates auxin homeostasis and male gametophyte development in Arabidopsis. Nat Commun..

[62] Lucca N, León G (2012). Arabidopsis *ACA7*, encoding a putative auto-regulated Ca^2+^-ATPase, is required for normal pollen development. Plant Cell Rep..

[63] Zhu J, Yuan S, Wei G, Qian D, Wu X, Jia H, Gui M, Liu W, An L, Xiang Y (2014). *Annexin5* is essential for pollen development in Arabidopsis. Mol Plant..

[64] Rotman N, Durbarry A, Wardle A, Yang WC, Chaboud A, Faure JE, Berger F, Twell D (2005). A Novel Class of MYB Factors Controls Sperm-Cell Formation in Plants. Curr Biol..

[65] Rieder B, Neuhaus HE (2011). Identification of an Arabidopsis Plasma Membrane-Located ATP Transporter Important for Anther Development. Plant Cell..

[66] Ishiguro S, Kawai-Oda A, Ueda J, Nishida I, Okada K (2001). The *DEFECTIVE IN ANTHER DEHISCIENCE* gene encodes a novel phospholipase A1 catalyzing the initial step of jasmonic acid biosynthesis, which synchronizes pollen maturation, anther dehiscence, and flower opening in Arabidopsis. Plant Cell..

[67] Peng J, Ilarslan H, Wurtele ES, Bassham DC (2011). *AtRabD2b* and *AtRabD2c* have overlapping functions in pollen development and pollen tube growth. BMC Plant Biol..

[68] Zhan H, Zhong Y, Yang Z, Xia H (2015). Enzyme activities of Arabidopsis inositol polyphosphate kinases AtIPK2α and AtIPK2β are involved in pollen development, pollen tube guidance and embryogenesis. Plant J..

[69] Adamczyk BJ, Fernandez DE (2009). MIKC* MADS domain heterodimers are required for pollen maturation and tube growth in Arabidopsis. Plant Physiol..

[70] Golovkin M, Reddy AS (2003). A calmodulin-binding protein from Arabidopsis has an essential role in pollen germination. Proc Natl Acad Sci USA..

[71] Wang SS, Diao WZ, Yang X, Qiao Z, Wang M, Acharya BR, Zhang W (2015). *Arabidopsis thaliana CML25* mediates the Ca^2+^ regulation of K^+^ transmembrane trafficking during pollen germination and tube elongation. Plant Cell Environ..

[72] Niu QK, Liang Y, Zhou JJ, Dou XY, Gao SC, Chen LQ, Zhang XQ, Ye D (2013). *Pollen-Expressed Transcription Factor 2* Encodes a Novel Plant-Specific TFIIB-Related Protein that Is Required for Pollen Germination and Embryogenesis in Arabidopsis. Mol Plant..

[73] Wang S, Okamoto T (2009). Involvement of polypyrimidine tract-binding protein (PTB)-related proteins in pollen germination in Arabidopsis. Plant Cell Physiol..

[74] Harrison-Lowe NJ, Olsen LJ (2008). *Autophagy protein 6* (*ATG6*) is required for pollen germination in *Arabidopsis thaliana*. Autophagy..

[75] Lalanne E, Honys D, Johnson A, Borner GHH, Lilley KS, Dupree P, Grossniklaus U, Twell D (2004). SETH1 and SETH2, Two Components of the Glycosylphosphatidylinositol Anchor Biosynthetic Pathway, Are Required for Pollen Germination and Tube Growth in Arabidopsis. Plant Cell..

[76] Palanivelu R, Brass L, Edlund AF, Preuss D (2003). Pollen tube growth and guidance is regulated by *POP2*, an Arabidopsis gene that controls GABA levels. Cell..

[77] Hoedemaekers K, Derksen J, Hoogstrate SW, Wolters-Arts M, Oh SA, Twell D, Mariani C, Rieu I (2015). *BURSTING POLLEN* is required to organize the pollen germination plaque and pollen tube tip in *Arabidopsis thaliana*. New Phytol..

[78] Liu XL, Liu L, Niu QK, Xia C, Yang KZ, Li R, Chen LQ, Zhang XQ, Zhou Y, Ye D (2011). *Male gametophyte defective 4* encodes a rhamnogalacturonan II xylosyltransferase and is important for growth of pollen tubes and roots in Arabidopsis. Plant J..

[79] Chang F, Gu Y, Ma H, Yang Z (2013). AtPRK2 Promotes ROP1 Activation via RopGEFs in the Control of Polarized Pollen Tube Growth. Mol Plant..

[80] Dai XR, Gao XQ, Chen GH, Tang LL, Wang H, Zhang XS (2014). APTG1, an ER-localized Mannosyltransferase Homolog of GPI10 in Yeast and PIG-B in Human, Is Required for Arabidopsis Pollen Tube Micropylar Guidance and Embryo Development. Plant Physiol..

[81] Zhang Y, He J, Mccormick S (2009). Two Arabidopsis AGC kinases are critical for the polarized growth of pollen tubes. Plant J..

[82] Birke H, Heeg C, Wirtz M, Hell R (2013). Successful Fertilization Requires the Presence of at Least One Major O-Acetylserine(thiol)lyase for Cysteine Synthesis in Pollen of Arabidopsis. Plant Physiol..

[83] Gu LL, Gao QF, Wang YF (2016). *Cyclic nucleotide-gated channel 18* is essential for pollen germination and pollen tube growth in Arabidopsis. Plant Signal Behav..

[84] Mouline K, Véry AA, Gaymard F, Boucherez J, Pilot G, Devic M, Bouchez D, Thibaud JB, Sentenac H (2002). Pollen tube development and competitive ability are impaired by disruption of a Shaker K^+^ channel in Arabidopsis. Genes Dev..

[85] Gutermuth T, Lassig R, Portes MT, Maierhofer T, Romeis T, Borst JW, Hedrich R, Feijó JA, Kai RK (2013). Pollen Tube Growth Regulation by Free Anions Depends on the Interaction between the Anion Channel SLAH3 and Calcium-Dependent Protein Kinases CPK2 and CPK20. Plant Cell..

[86] Lu Y, Chanroj S, Zulkifli L, Johnson MA, Uozumi N, Cheung A, Sze H (2011). Pollen tubes lacking a pair of K^+^ transporters fail to target ovules in Arabidopsis. Plant Cell..

[87] Zhou L, Lan W, Chen B, Fang W, Luan S (2015). A Calcium Sensor-Regulated Protein Kinase CIPK19 Is Required for Pollen Tube Growth and Polarity. Plant Physiol..

[88] Berkowitz O, Jost R, Pollmann S, Masle J (2008). Characterization of TCTP, the Translationally Controlled Tumor Protein, from *Arabidopsis thaliana*. Plant Cell..

[89] Madison SL, Buchanan ML, Glass JD, Mcclain TF, Park E, Nebenführ A (2015). Class XI Myosins Move Specific Organelles in Pollen Tubes and Are Required for Normal Fertility and Pollen Tube Growth in Arabidopsis. Plant Physiol..

[90] Alandete-Saez M, Ron M, McCormick S (2008). *GEX3*, Expressed in the Male Gametophyte and in the Egg Cell of *Arabidopsis thaliana*, Is Essential for Micropylar Pollen Tube Guidance and Plays a Role during Early Embryogenesis. Mol Plant..

[91] Dong X, Hong Z, Sivaramakrishnan M, Mahfouz M, Verma DP (2005). *Callose synthase* (*CalS5*) is required for exine formation during microgametogenesis and for pollen viability in Arabidopsis. Plant J..

[92] Hird DL, Worrall D, Hodge R, Smartt S, Paul W, Scott R (1993). The anther-specific protein encoded by the *Brassica napus* and *Arabidopsis thaliana A6* gene displays similarity to beta-1,3-glucanases. Plant J..

[93] Wan L, Zha W, Cheng X, Liu C, Lv L, Liu C, Wang Z, Du B, Chen R, Zhu L, He G (2011). A rice beta-1, 3-glucanase gene *Osg1* is required for callose degradation in pollen development. Planta..

[94] Lu P, Chai M, Yang J, Ning G, Wang G, Ma H (2014). The Arabidopsis *CALLOSE DEFECTIVE MICROSPORE1* gene is required for male fertility through regulating callose metabolism during microsporogenesis. Plant Physiol..

[95] Doxey AC, Yaish MW, Moffatt BA, Griffith M, McConkey BJ (2007). Functional divergence in the Arabidopsis beta-1,3-glucanase gene family inferred by phylogenetic reconstruction of expression states. Mol Biol Evol..

[96] Quilichini TD, Douglas CJ, Samuels AL (2014). New views of tapetum ultrastructure and pollen exine development in *Arabidopsis thaliana*. Ann Bot..

[97] Ting JTL, Wu SSH, Ratnayake C, Huang AHC (1998). Constituents of the tapetosomes and elaioplasts in *Brassica campestris* tapetum and their degradation and retention during microsporogenesis. Plant J..

[98] Hsieh K, Huang AH (2005). Lipid-rich tapetosomes in Brassica tapetum are composed of oleosin-coated oil droplets and vesicles, both assembled in and then detached from the endoplasmic reticulum. Plant J..

[99] Xu J, Ding Z, Vizcay-Barrena G, Shi J, Liang W, Yuan Z, Werck-Reichhart D, Schreiber L, Wilson ZA, Zhang D (2014). ABORTED MICROSPORES Acts as a Master Regulator of Pollen Wall Formation in Arabidopsis. Plant Cell..

[100] Dun X, Zhou Z, Xia S, Wen J, Yi B, Shen J, Ma C, Tu J, Fu T (2011). BnaC.Tic40, a plastid inner membrane translocon originating from *Brassica oleracea*, is essential for tapetal function and microspore development in *Brassica napus*. Plant J..

[101] Song L, Zhou Z, Tang S, Zhang Z, Xia S, Qin M, Li B, Wen J, Yi B, Shen J, Ma C, Fu T, Tu J (2016). Ectopic Expression of BnaC.CP20.1 Results in Premature Tapetal Programmed Cell Death in Arabidopsis. Plant Cell Physiol..

[102] Dong X, Feng H, Xu M, Lee J, Kim YK, Lim YP, Piao Z, Park YD, Ma H, Hur Y (2013). Comprehensive analysis of genic male sterility-related genes in *Brassica rapa* using a newly developed Br300K oligomeric chip. PloS one..

[103] Liu C, Liu Z, Li C, Zhang Y, Feng H (2016). Comparative transcriptome analysis of fertile and sterile buds from a genetically male sterile line of Chinese cabbage. In Vitro Cell Dev Biol Plant..

[104] Qu C, Fu F, Liu M, Zhao H, Liu C, Li J, Tang Z, Xu X, Qiu X, Wang R, Lu K (2015). Comparative Transcriptome Analysis of Recessive Male Sterility (RGMS) in Sterile and Fertile *Brassica napus* Lines. PloS one..

[105] Qin Y, Leydon AR, Manziello A, Pandey R, Mount D, Denic S, Vasic B, Johnson MA, Palanivelu R (2009). Penetration of the stigma and style elicits a novel transcriptome in pollen tubes, pointing to genes critical for growth in a pistil. PLoS Genet..

[106] Wolf S, Mouille G, Pelloux J (2009). Homogalacturonan methyl-esterification and plant development. Mol Plant..

[107] Tian GW, Chen MH, Zaltsman A, Citovsky V (2006). Pollen-specific pectin methylesterase involved in pollen tube growth. Dev Biol..

[108] Yue X, Lin S, Yu Y, Huang L, Cao J (2018). The putative pectin methylesterase gene, *BcMF23a*, is required for microspore development and pollen tube growth in *Brassica campestris*. Plant Cell Rep..

[109] Wolf S, Grsic-Rausch S, Rausch T, Greiner S (2003). Identification of pollen-expressed pectin methylesterase inhibitors in Arabidopsis. FEBS Lett..

[110] Zhang GY, Feng J, Wu J, Wang XW (2010). BoPMEI1, a pollen-specific pectin methylesterase inhibitor, has an essential role in pollen tube growth. Planta..

[111] Woriedh M, Wolf S, Marton ML, Hinze A, Gahrtz M, Becker D, Dresselhaus T (2013). External application of gametophyte-specific ZmPMEI1 induces pollen tube burst in maize. Plant Reprod..

[112] Paynel F, Leroux C, Surcouf O, Schaumann A, Pelloux J, Driouich A, Mollet JC, Lerouge P, Lehner A, Mareck A (2014). Kiwi fruit PMEI inhibits PME activity, modulates root elongation and induces pollen tube burst in *Arabidopsis thaliana*. Plant Growth Regul..

[113] Huang L, Cao J, Zhang A, Ye Y, Zhang Y, Liu T (2009). The polygalacturonase gene *BcMF2* from *Brassica campestris* is associated with intine development. J Exp Bot..

[114] Huang L, Ye Y, Zhang Y, Zhang A, Liu T, Cao J (2009). *BcMF9*, a novel polygalacturonase gene, is required for both *Brassica campestris* intine and exine formation. Ann Bot..

[115] Zhang Q, Huang L, Liu T, Yu X, Cao J (2008). Functional analysis of a pollen-expressed polygalacturonase gene *BcMF6* in Chinese cabbage (*Brassica campestris* L. ssp. *chinensis* Makino). Plant Cell Rep..

[116] Yu Y, Lv M, Liang Y, Xiong X, Cao J (2014). Molecular Cloning and Characterization of a Novel Polygalacturonase Gene, *BcMF24*, Involved in Pollen Development of *Brassica campestris ssp. chinensis*. Plant Mol Biol Rep..

[117] Khurana R, Kapoor S, Tyagi AK (2012). Anthology of Anther/Pollen-Specific Promoters and Transcription Factors. Crit Rev Plant Sci..

[118] Jenuwein T (2006). The epigenetic magic of histone lysine methylation. FEBS J..

[119] Kim DH, Tang Z, Shimada M, Fierz B, Houck-Loomis B, Bar-Dagen M, Lee S, Lee SK, Muir TW, Roeder RG, Lee JW (2013). Histone H3K27 trimethylation inhibits H3 binding and function of SET1-like H3K4 methyltransferase complexes. Mol Cell Biol..

[120] Berr A, McCallum EJ, Menard R, Meyer D, Fuchs J, Dong A, Shen WH (2010). Arabidopsis *SET DOMAIN GROUP 2* is required for H3K4 trimethylation and is crucial for both sporophyte and gametophyte development. Plant Cell..

[121] Cartagena JA, Matsunaga S, Seki M, Kurihara D, Yokoyama M, Shinozaki K, Fujimoto S, Azumi Y, Uchiyama S, Fukui K (2008). The Arabidopsis *SDG4* contributes to the regulation of pollen tube growth by methylation of histone H3 lysines 4 and 36 in mature pollen. Dev Biol..

[122] Huang L, Dong H, Zhou D, Li M, Liu Y, Zhang F, Feng Y, Yu D, Lin S, Cao J (2018). Systematic identification of long non-coding RNAs during pollen development and fertilization in *Brassica rapa*. Plant J..

[123] Lin S, Dong H, Zhang F, Qiu L, Wang F, Cao J, Huang L (2014). *BcMF8*, a putative arabinogalactan protein-encoding gene, contributes to pollen wall development, aperture formation and pollen tube growth in *Brassica campestris*. Ann Bot..

[124] Tian T, Liu Y, Yan H, You Q, Yi X, Du Z, Xu W, Su Z (2017). AgriGO v2.0: a GO analysis toolkit for the agricultural community, 2017 update. Nucleic Acids Res..

[125] Ye J, Fang L, Zheng H, Zhang Y, Chen J, Zhang Z, Wang J, Li S, Li R, Bolund L, Wang J (2006). WEGO: a web tool for plotting GO annotations. Nucleic Acids Res..

[126] Moriya Y, Itoh M, Okuda S, Yoshizawa AC, Kanehisa M (2007). KAAS: an automatic genome annotation and pathway reconstruction server. Nucleic Acids Res..

[127] Livak KJ, Schmittgen TD (2001). Analysis of relative gene expression data using real-time quantitative PCR and the 2^-△△CT^ Method. Methods..

[128] Clough SJ, Bent AF (1998). Floral dip: a simplified method for *Agrobacterium*-mediated transformation of *Arabidopsis thaliana*. Plant J..

[129] Kim YY, Choi H, Segami S, Cho HT, Martinoia E, Maeshima M, Lee Y (2009). *AtHMA1* contributes to the detoxification of excess Zn(II) in Arabidopsis. Plant J..

[130] Wamstad JA, Alexander JM, Truty RM, Shrikumar A, Li F, Eilertson KE, Ding H, Wylie JN, Pico AR, Capra JA, Erwin G, Kattman SJ, Keller GM, Srivastava D, Levine SS, Pollard KS, Holloway AK, Boyer LA, Bruneau BG (2012). Dynamic and coordinated epigenetic regulation of developmental transitions in the cardiac lineage. Cell..

[131] Zhang Y, Liu T, Meyer CA, Jérôme E, Johnson DS, Bernstein BE, Chad N, Myers RM, Myles B, Li W (2008). Model-based Analysis of ChIP-Seq (MACS). Genome Biol..

[132] Quinlan AR, Hall IM (2010). BEDTools: a flexible suite of utilities for comparing genomic features. Bioinformatics..

