# Supplementary figures and images for: Comparative transcriptome analysis and ChIP-sequencing reveals stage-specific gene expression and regulation profiles associated with pollen wall formation in Brassica rapa
Source: BMC Genomics. 2019 Apr 3;20:264. doi: 10.1186/s12864-019-5637-x (PMC6446297; doi:10.1186/s12864-019-5637-x)

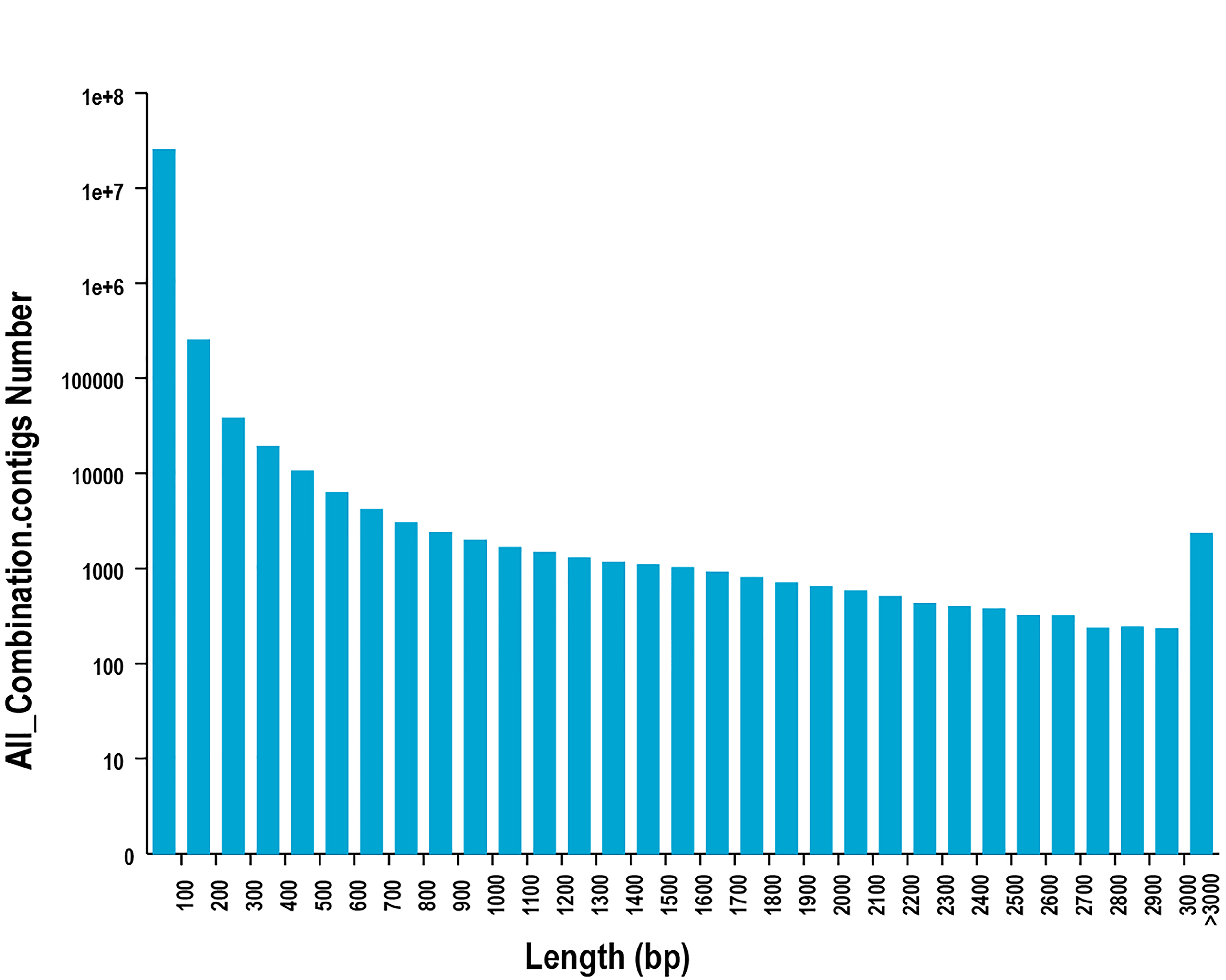

Supplement: Supplementary file 1 — Figure S1. The length distribution of assembled Brassica rapa contigs. (TIF 1833 kb) [file 12864_2019_5637_MOESM1_ESM.tif]

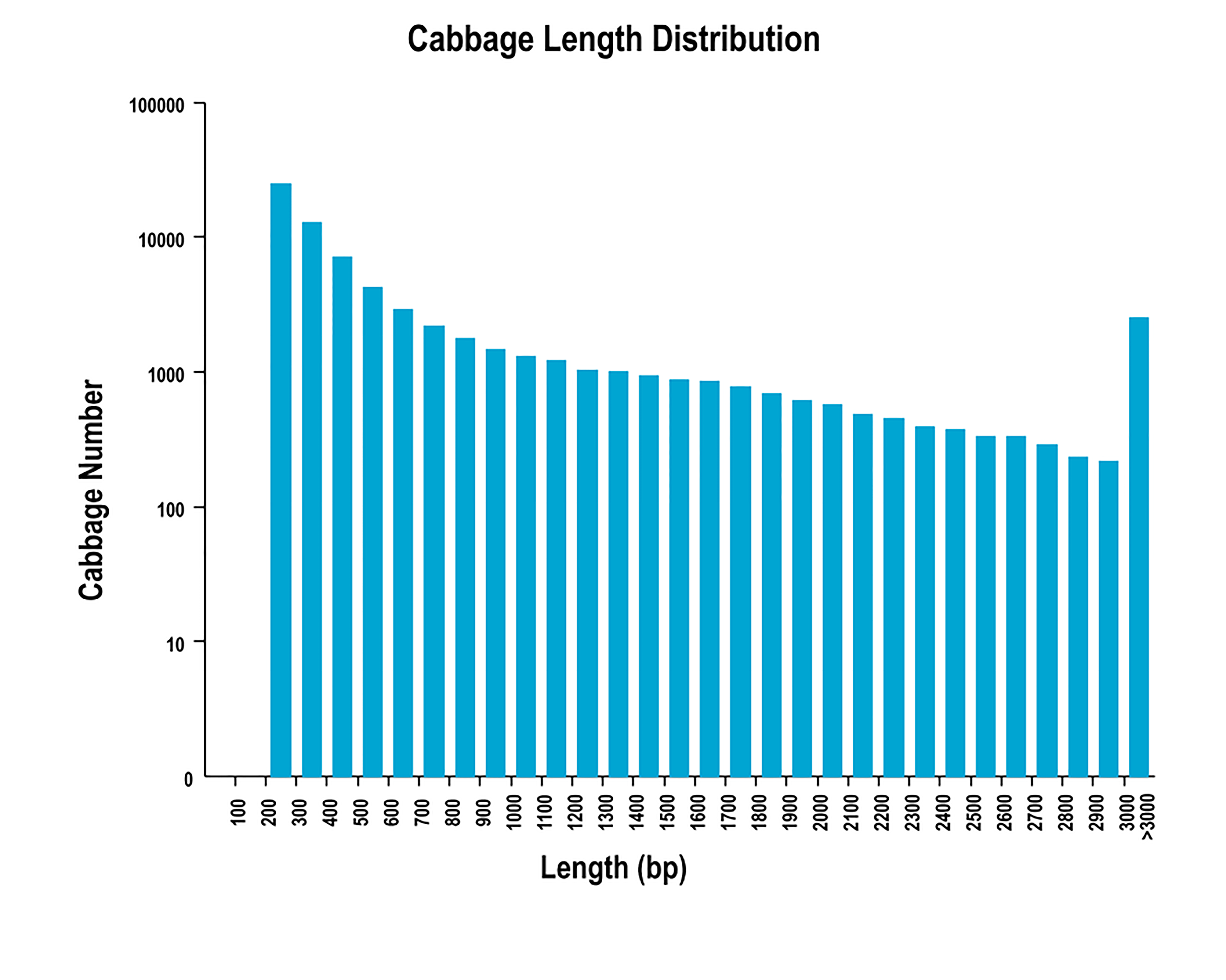

Supplement: Supplementary file 3 — Figure S2. The length distribution of assembled Brassica rapa Unigenes. (TIF 2354 kb) [file 12864_2019_5637_MOESM3_ESM.tif]

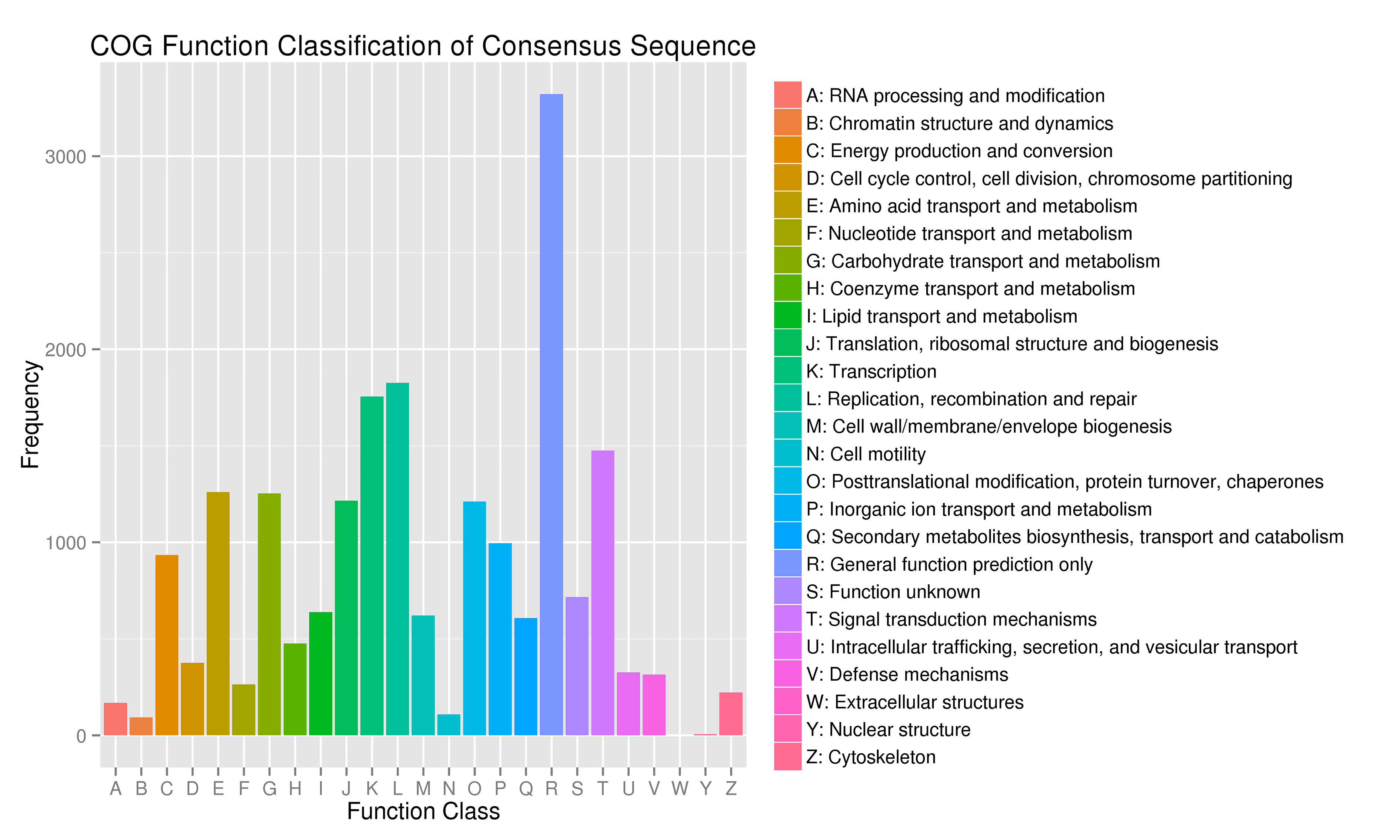

Supplement: Supplementary file 4 — Figure S3. COG functional classification of all Unigene sequences. (TIF 1450 kb) [file 12864_2019_5637_MOESM4_ESM.tif]

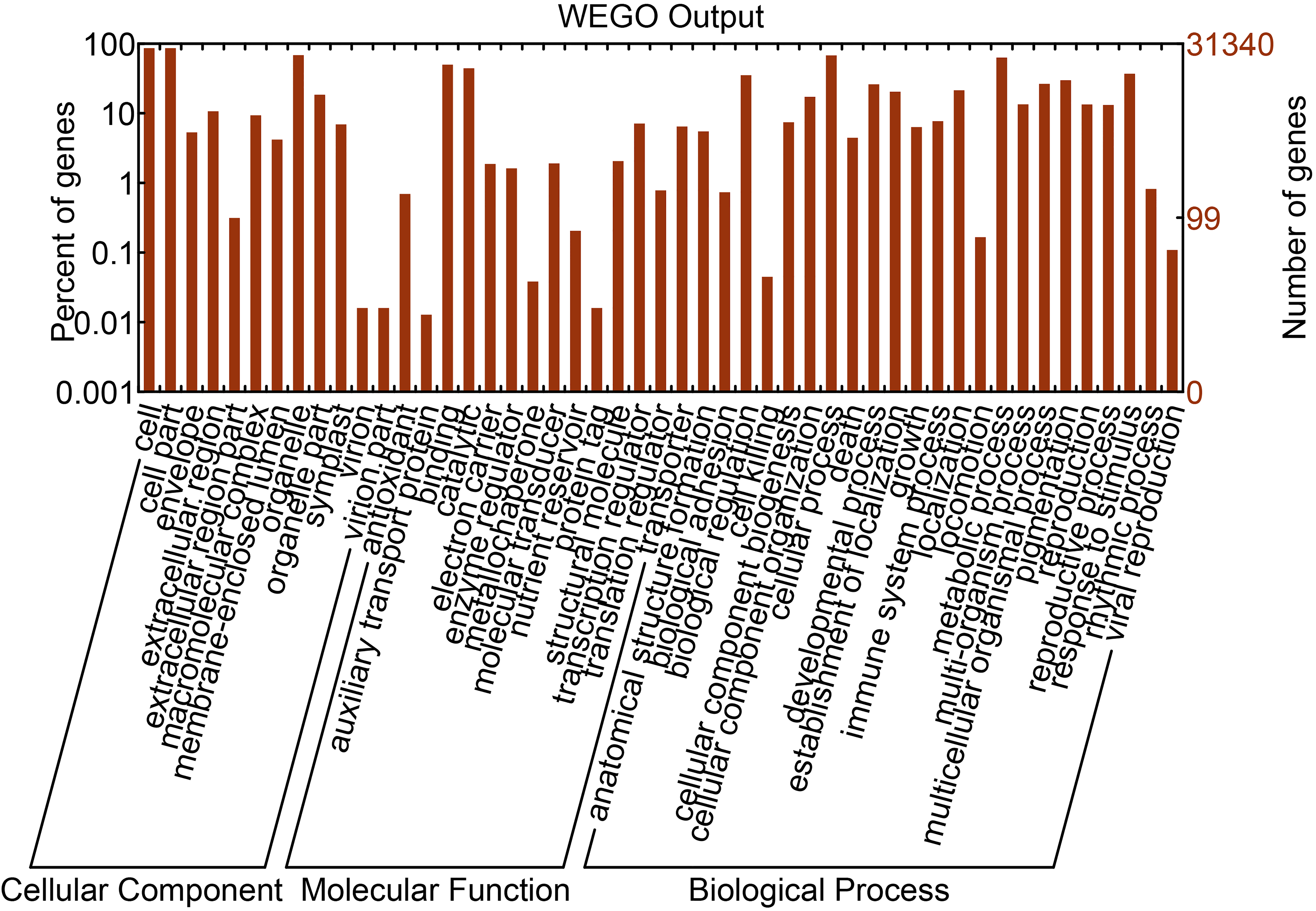

Supplement: Supplementary file 5 — Figure S4. GO classification of Brassica rapa Unigenes. (TIF 5347 kb) [file 12864_2019_5637_MOESM5_ESM.tif]

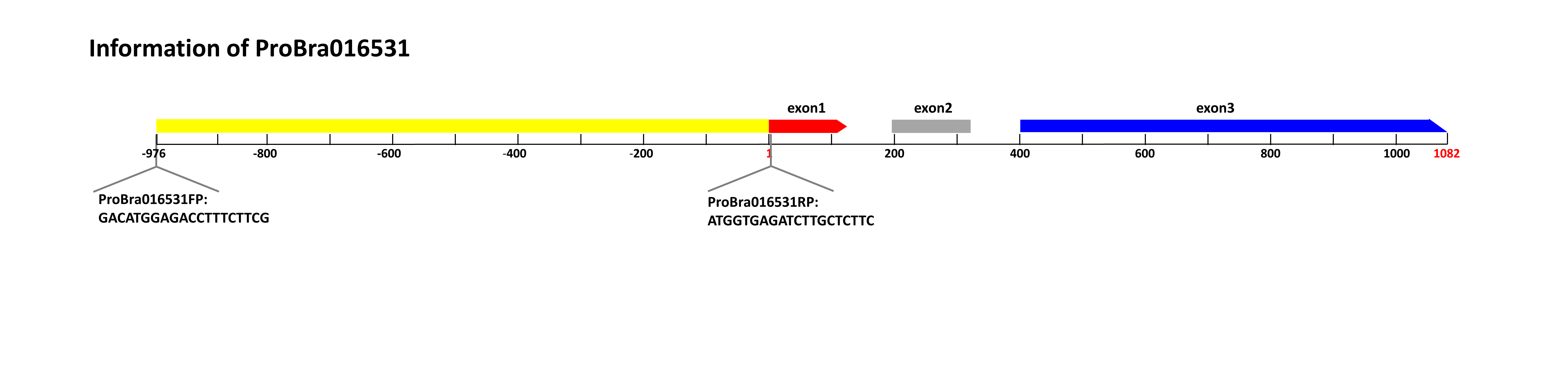

Supplement: Supplementary file 6 — Figure S5. Information of ProBra016531. (TIF 464 kb) [file 12864_2019_5637_MOESM6_ESM.tif]

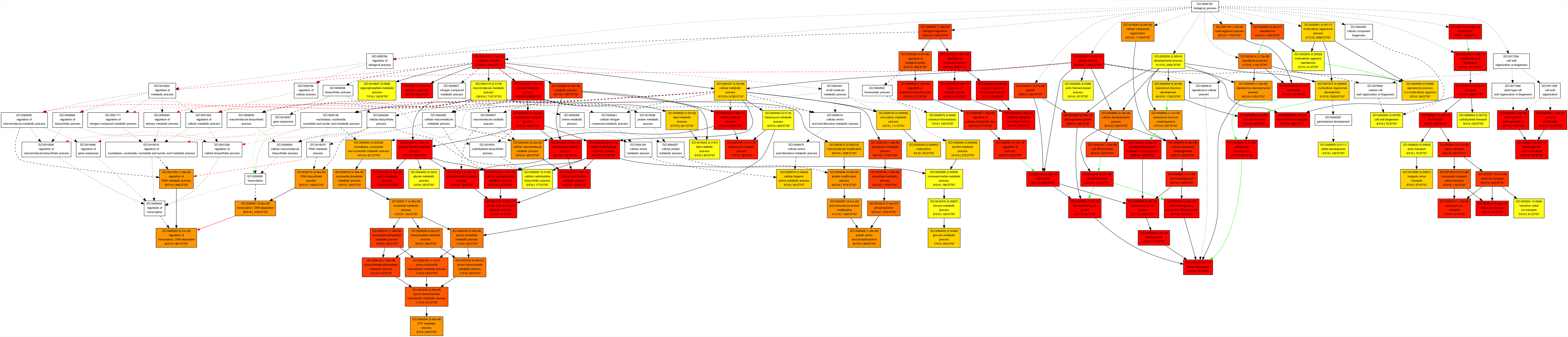

Supplement: Supplementary file 7 — Figure S6. Significantly enriched Biological Processes GO terms in the group of down-regulated genes marked with H3K27me3. (TIF 2813 kb) [file 12864_2019_5637_MOESM7_ESM.tif]

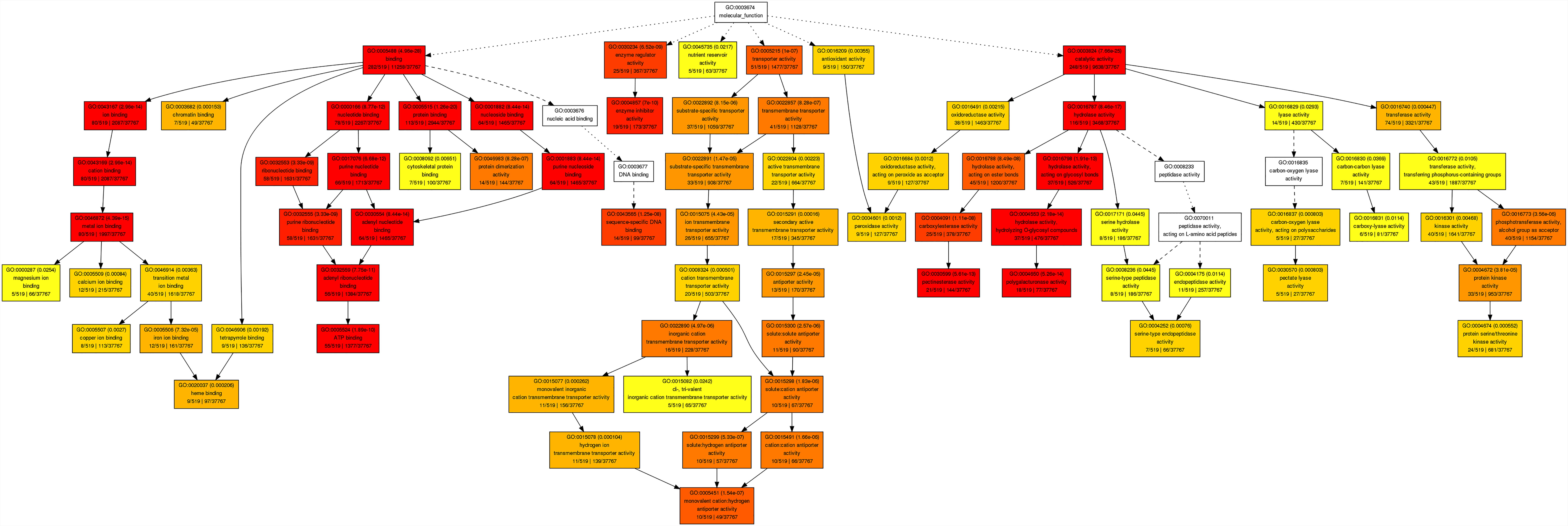

Supplement: Supplementary file 8 — Figure S7. Significantly enriched Molecular Functions GO terms in the group of down-regulated genes marked with H3K27me3. (TIF 4779 kb) [file 12864_2019_5637_MOESM8_ESM.tif]
